# Estimation of unconfirmed COVID-19 cases from a cross-sectional survey of >10 000 households and a symptom-based machine learning model in Gilgit-Baltistan, Pakistan

**DOI:** 10.1136/bmjph-2024-001255

**Published:** 2025-04-28

**Authors:** Daniel S Farrar, Lisa G Pell, Yasin Muhammad, Sher Hafiz Khan, Lauren Erdman, Diego G Bassani, Zachary Tanner, Imran Ahmed Chauhadry, Muhammad Karim, Falak Madhani, Shariq Paracha, Masood Ali Khan, Sajid Soofi, Monica Taljaard, Rachel F Spitzer, Sarah M Abu Fadaleh, Zulfiqar A Bhutta, Shaun K Morris

**Affiliations:** 1 Centre for Global Child Health, The Hospital for Sick Children, Toronto, Ontario, Canada; 2 Gilgit Regional Office, Aga Khan Health Service Pakistan, Gilgit, Gilgit-Baltistan, Pakistan; 3 Vector Institute, The Hospital for Sick Children and University of Toronto, Toronto, Ontario, Canada; 4 Cincinnati Children’s Hospital Medical Center, Cincinnati, Ohio, USA; 5 Department of Pediatrics, Temerty Faculty of Medicine, University of Toronto, Toronto, Ontario, Canada; 6 Dalla Lana School of Public Health, University of Toronto, Toronto, Ontario, Canada; 7 Centre of Excellence in Women and Child Health, The Aga Khan University, Karachi, Sindh, Pakistan; 8 Aga Khan Health Service Pakistan, Karachi, Sindh, Pakistan; 9 Brain and Mind Institute, The Aga Khan University, Karachi, Sindh, Pakistan; 10 Clinical Epidemiology Program, Ottawa Health Research Institute, Ottawa, Ontario, Canada; 11 School of Epidemiology and Public Health, University of Ottawa, Ottawa, Ontario, Canada; 12 Department of Obstetrics and Gynaecology, University of Toronto, Toronto, Ontario, Canada; 13 Section of Gynecology, The Hospital for Sick Children, Toronto, Ontario, Canada; 14 Institute for Global Health & Development, The Aga Khan University, South-Central Asia & East Africa, Pakistan; 15 Division of Infectious Diseases, The Hospital for Sick Children, Toronto, Ontario, Canada

**Keywords:** COVID-19, Epidemiology, Cross-Sectional Studies, Statistics as Topic, Population Surveillance

## Abstract

**Introduction:**

Robust estimates of COVID-19 prevalence in settings with limited capacity for SARS-CoV-2 molecular and serologic testing are scarce. We aimed to describe the epidemiology of confirmed and probable COVID-19 in Gilgit-Baltistan, and to develop a symptom-based predictive model to identify infected but undiagnosed individuals with COVID-19.

**Methods:**

We conducted a cross-sectional survey in 10 257 randomly selected households in Gilgit-Baltistan from June to August 2021. Data regarding SARS-CoV-2 testing, healthcare worker (HCW) diagnoses, symptoms and outcomes since March 2020 were self-reported by households. ‘Confirmed/probable’ infection was defined as a positive test, HCW COVID-19 diagnosis or HCW pneumonia diagnosis with COVID-19-positive contact. Robust Poisson regression was conducted to assess differences in symptoms, outcomes and SARS-CoV-2 testing rates. We developed a symptom-based machine learning model to differentiate confirmed/probable infections from those with negative tests. We applied this model to untested respondents to estimate the total prevalence of SARS-CoV-2 infection.

**Results:**

Data were collected for 77 924 people. Overall, 314 (0.5%) had confirmed/probable infections, 3263 (4.4%) had negative tests and 74 347 (95.1%) were untested. Children were tested less often than adults (adjusted prevalence ratio (aPR) 0.08, 95% CI 0.06 to 0.12 for ages 1–4 years vs 30–39 years), while males were tested more often than females (aPR 1.51, 95% CI 1.40 to 1.63). In the predictive model, area under the receiver operating characteristic curve was 0.92 (95% CI 0.90 to 0.93). We estimate there were 8–17 total SARS-CoV-2 infections for each positive test (8–17:1). The ratio of estimated to confirmed cases was higher for ages 1–4 years (211–480:1), 5–9 years (80–185:1) and for females (13–25:1).

**Conclusions:**

From March 2020 to August 2021, the majority of SARS-CoV-2 infections in Gilgit-Baltistan went unconfirmed, particularly among women and children. Predictive models which incorporate self-reported symptoms may improve understanding of the burden of disease in settings lacking diagnostic capacity.

**WHAT IS ALREADY KNOWN ON THIS TOPIC**

- The total prevalence of SARS-CoV-2 in Gilgit-Baltistan and other settings with limited laboratory testing capacity remains unknown.
- Few representative studies describing the epidemiology and clinical features of COVID-19 have been conducted in Gilgit-Baltistan.
- Strategies to determine the true prevalence of COVID-19 have included compartmental models, machine learning, seroprevalence surveys and excess mortality.

**WHAT THIS STUDY ADDS**

- In Gilgit-Baltistan, the total estimated prevalence of SARS-CoV-2 infection was 8–17 times greater than the number of test-positive individuals.
- Rates of SARS-CoV-2 testing were 34% lower in females versus males, and 92% less in ages 1–4 years vs ages 30–39 years. SARS-CoV-2 underdetection was therefore more common in these populations (13–25 total cases per test-positive female; 211–480 total cases per test-positive child aged 1–4 years).
- In the absence of testing, healthcare worker diagnoses of COVID-19 and pneumonia/lung infection were common.
- Respiratory viruses other than SARS-CoV-2 were much less prevalent during the study recall period, increasing the likelihood that predicted infections were attributable to SARS-CoV-2.

**HOW THIS STUDY MIGHT AFFECT RESEARCH, PRACTICE OR POLICY**

- The true prevalence of SARS-CoV-2 infection in resource-limited settings is likely to be substantially greater than the number of positive SARS-CoV-2 tests reported by government public health institutions.
- Symptom-based modelling which incorporates primary data may serve as an alternative to seroprevalence studies when attempting to understand the disease epidemiology of SARS-CoV-2 and may have utility for future respiratory viral outbreaks and potentially other disease entities.
- Gender-based and age-based disparities in availability of testing should be considered when evaluating the burden of COVID-19 and can inform the need for preventive and treatment modalities in future waves of SARS-CoV-2 infection.

## Introduction

 Robust estimates of the prevalence of SARS-CoV-2 infection during the COVID-19 pandemic remain unknown for many regions globally, including Pakistan. Previous studies have estimated a wide range in the proportion of SARS-CoV-2 infections which test positive from all incident infections, between 2% and 100% across countries in 2020.^1^,^3^ From 2020–2022, when routine SARS-CoV-2 testing was relatively common globally, the undercounting of SARS-CoV-2 infections was attributable to multiple factors including limited availability of diagnostic testing, a high proportion of asymptomatic or mildly symptomatic presentations and individuals not presenting to healthcare infrastructure.^4^ Substantially more uncounted infections may be expected in low-resource settings, where laboratory and diagnostic capacity is lacking. Novel approaches are, therefore, needed to improve understanding of the burden of SARS-CoV-2 in remote populations, to inform public health decision-making including healthcare and laboratory resource allocation, and to infer acute and longer-term sequelae of SARS-CoV-2.

In Gilgit-Baltistan, a remote and mountainous administrative territory in northern Pakistan, regular access to healthcare services is limited by long distances to healthcare facilities, underdeveloped road infrastructure and extreme weather conditions including landslides and seasonal snowfall.^5 6^ The majority of primary healthcare services are delivered by Lady Health Workers, who are community-based and government-sponsored health workers who provide routine health education, basic preventive and curative care, and referral for serious health conditions.^7^ However, access to diagnostic and laboratory services is scarce, including for SARS-CoV-2.^8^

As of 13 June 2021, 5712 confirmed SARS-CoV-2 infections and 108 confirmed COVID-19-related deaths had been reported in Gilgit-Baltistan, among a population of approximately 1.8 million people.^9^ Prior to this date, 147 daily molecular tests were conducted on average, equivalent to 0.1 daily tests per 1000 population (online supplemental appendix 1). This per-capita testing rate was similar to all Pakistan (0.1 daily tests per 1000) but lower than most countries during the same period, such as Iran (0.6 daily tests per 1000), India (0.6 daily tests per 1000) and the USA (2.9 daily tests per 1000).^10^ The true burden of SARS-CoV-2 infection in Gilgit-Baltistan, therefore, remains unknown.

In this study, we conducted a cross-sectional survey of 10 257 households in Gilgit-Baltistan to collect detailed information on COVID-19-related symptoms, diagnoses and outcomes. First, we aimed to describe the epidemiology and clinical features of confirmed and probable COVID-19 in the population. Second, given the low rate of SARS-CoV-2 testing in Gilgit-Baltistan, we developed a predictive model using machine learning to identify infected but untested or undiagnosed individuals with COVID-19. Machine learning has previously been used to predict SARS-CoV-2 infection and death^11^,^15^ and offers an alternative mechanism to directly estimate the total prevalence of SARS-CoV-2 infection over more costly or indirect methods including seroprevalence surveys,^16^ compartmental models^17^ and excess mortality.^18^ Through the combination of epidemiological data and machine learning, we aim to inform the true burden of SARS-CoV-2 infection in Gilgit-Baltistan and settings with similar diagnostic testing limitations.

## Materials and methods

### Study design and procedures

We conducted a cross-sectional household survey in 7 (of 10) districts of Gilgit-Baltistan, Pakistan. Gilgit-Baltistan has a population of approximately 1.8 million people, with more than half aged <20 years, and the majority of whom lives in remote villages within valleys of the Karakoram and Himalaya mountain ranges.^19^ The survey was conducted from 14 June 2021 to 31 August 2021, during the baseline phase of a cluster-randomised controlled trial (ClinicalTrials.gov NCT04798833), which aims to estimate the effect of an integrated newborn care kit on the all-cause neonatal mortality rate (NMR).^20^ The primary objective of this baseline data collection phase was to describe NMR and other newborn and maternal outcomes in the study catchment area prior to the introduction of the trial’s intervention and to calculate inputs for the trial’s covariate constrained randomisation and prespecified multivariable analyses. We leveraged this existing survey to conduct a second, unrelated survey collecting information regarding COVID-19. Further details of the trial and baseline maternal and newborn outcomes are described elsewhere.^20 21^

The household survey was conducted using a two-stage, stratified sampling design. To match the trial, the sampling frame was limited to villages with pre-existing Lady Health Worker coverage, and participant selection was stratified by the 77 available trial clusters. Clusters were defined as Union Councils (or sub-Union Councils), which are district subdivisions that facilitate delivery of local services including healthcare, education and sanitation. Clusters from all seven districts (Astore, Diamer, Ghanche, Kharmang, Nagar, Shigar and Skardu) were included. The first sampling stage was the selection of villages (the primary sampling unit), and three villages per stratum were selected with a probability proportional to village population size. Data collection teams then visited each selected village to conduct a standard household listing operation to identify and map occupied households. Specifically, data collectors toured each selected village to understand its boundaries, layout and approximate size. Villages with >300 estimated dwellings were segmented into approximately equal sizes and randomly selected as above. Data collectors then enumerated all free-standing residential structures in the village or segment, excluding non-residential, abandoned or empty households from the list of households eligible for selection. Household listing procedures were based on guidelines and documents from the US Agency for International Development.^22^ On completion of household listing, 48 households (the secondary sampling unit) were selected per village with an equal probability systematic selection approach. If <48 occupied households were identified in a village, all households were contacted for participation in the survey. The sample size for the household survey was determined for the primary outcome of neonatal mortality, using an assumed NMR of 35 per 1000 live births, precision of 6 per 1000 live births and design effect of 2.03.^23^ Assuming a similar response rate as the 2017–2018 Pakistan Demographic and Health Survey, we aimed to enrol 11 231 households.

Selected households were visited by trained data collectors to obtain verbal informed consent and to administer the survey using a structured questionnaire. For field team safety, households found to be at elevated risk for active SARS-CoV-2 infection (ie, diagnosed with COVID-19 or asked to self-isolate in the past 14 days) were not eligible to participate. As such, we describe only prior (and not current) SARS-CoV-2 infections in this study. Consenting participants, most commonly the head of the household, were asked to self-report all elements of the questionnaire on behalf of the household. Following the primary series of questions regarding maternal and newborn outcomes, we collected individual-level information for each household member regarding symptoms, outcomes including hospitalisation or death and any close contact with individuals diagnosed with COVID-19 (online supplemental appendix 2). To ascertain a history of previous SARS-CoV-2 infection confirmed through either virologic or serologic testing, we asked about any SARS-CoV-2 testing (either nasal swab, oral swab or blood draw) and the results of any reported tests regardless of modality. If no positive test was reported, respondents were asked if a healthcare worker (HCW) had ever diagnosed the individual with either COVID-19 or ‘pneumonia/lung infection’ without conducting a test. The recall period for all COVID-19-related questions was between March 2020 (ie, beginning of the COVID-19 pandemic in Pakistan) and the date of interview.

### Study definitions

All individuals were categorised into one of five mutually exclusive groups on the basis of certainty of prior SARS-CoV-2 infection. Known SARS-CoV-2 history included: (1) Confirmed or probable infection, defined as having a positive SARS-CoV-2 test, HCW diagnosis of COVID-19 or HCW diagnosis of pneumonia/lung infection with a COVID-19 positive close contact and (2) Negative SARS-CoV-2, defined as any individual with a negative test and lacking HCW diagnoses of COVID-19 or pneumonia/lung infection with positive close contact. Untested individuals with unknown SARS-CoV-2 history were categorised as (3) those with COVID-19 positive close contacts but without HCW diagnoses of pneumonia/lung infection; (4) those with HCW diagnoses of pneumonia/lung infection but without COVID-19 positive close contacts and (5) those with completely unknown history (ie, without SARS-CoV-2 testing, HCW diagnoses or known close contacts). Close contacts were defined as any household or external contact with whom they shared an enclosed, indoor space within six feet for at least 15 minutes and who had tested positive or been diagnosed with COVID-19 in the 2 weeks prior to an individual’s reported symptoms.

### Statistical analysis

Household and individual characteristics were summarised using unweighted counts, weighted percentages and weighted medians and IQRs. Household weights were calculated using templates from the Multiple Indicator Cluster Surveys^24^ and accounted for the two-stage sampling design as well as household non-response and were applied to all analyses using the Stata svyset command. Age-adjusted and sex-adjusted robust Poisson regression was conducted to compare characteristics between: (a) individuals with confirmed/probable infection vs negative SARS-CoV-2 tests; (b) individuals tested vs not tested for SARS-CoV-2 and (c) individuals with confirmed/probable infection by disease severity (ie, hospitalised/deceased vs not hospitalised/deceased). Associations were reported using adjusted prevalence ratios (aPR) and 95% CIs. We further assessed testing biases by dividing the number of age-specific and sex-specific completed tests by the corresponding sample denominators. To gauge the representativeness of the sample population, we compared study SARS-CoV-2 testing, case and positivity rates to statistics reported by the Gilgit-Baltistan Department of Health. All analyses were conducted using a complete case approach, with the frequency of missing data reported in footnotes. Tests of association were assessed at the α=0.05 significance level and did not account for multiple statistical testing.

Given the low frequency of testing in this population, we developed a machine learning model to predict prior SARS-CoV-2 infection among individuals with known SARS-CoV-2 histories (ie, confirmed/probable infection vs negative SARS-CoV-2). Here, we used extreme gradient boosting (XGBoost)^25^ and bootstrap validation, with decision trees constructed using available data regarding age (analysed as a restricted cubic spline with four knots), sex, 11 binary presenting symptoms, any healthcare seeking (vs none), a composite variable denoting hospitalisation or death (vs neither) and a village-level variable denoting the Z-score normalised rate of confirmed/probable infections. Details on model selection, hyperparameters and validation are available in online supplemental appendix 3. We report validation statistics and bootstrapped CIs for the XGBoost model, including area under the receiver operating characteristic curve (auROC), area under the precision-recall curve (auPRC), calibration curves, sensitivity and specificity.

The validated XGBoost model was applied to all individuals with unknown SARS-CoV-2 history to predict prior SARS-CoV-2 diagnosis. The number of predicted cases was summarised using frequencies and bootstrapped 95% CIs. We then compared the number of individuals with positive SARS-CoV-2 tests (ie, those who would be reported through government public health institutions) to the number of probable cases (including HCW diagnoses or predicted from the XGBoost model) and possible cases (including HCW diagnoses of ‘pneumonia/lung infection’ and individuals with COVID-19 positive close contacts). Comparisons were drawn overall and by age group, sex and study district.

Results were reported in accordance with the STROBE^26^ and TRIPOD+AI^27^ checklists. Descriptive analyses were conducted using Stata, V.18.0,^28^ while machine learning analyses were conducted in Python using the scikit-learn library.^29^

### Patient and public involvement

No patients were involved in conducting this research. Public involvement with representatives of the Gilgit-Baltistan Department of Health was sought during the study design phase of the cluster randomised trial, to ensure the study aligned with local health priorities. Letters of Cooperation were signed by the Gilgit-Baltistan Secretary of Health, following unanimous approval by review committee members. Our study team also engaged in informal meetings with community members, religious leaders and lambardars to ensure community engagement and understanding for the study.

## Results

### Population description

**Figure 1:**
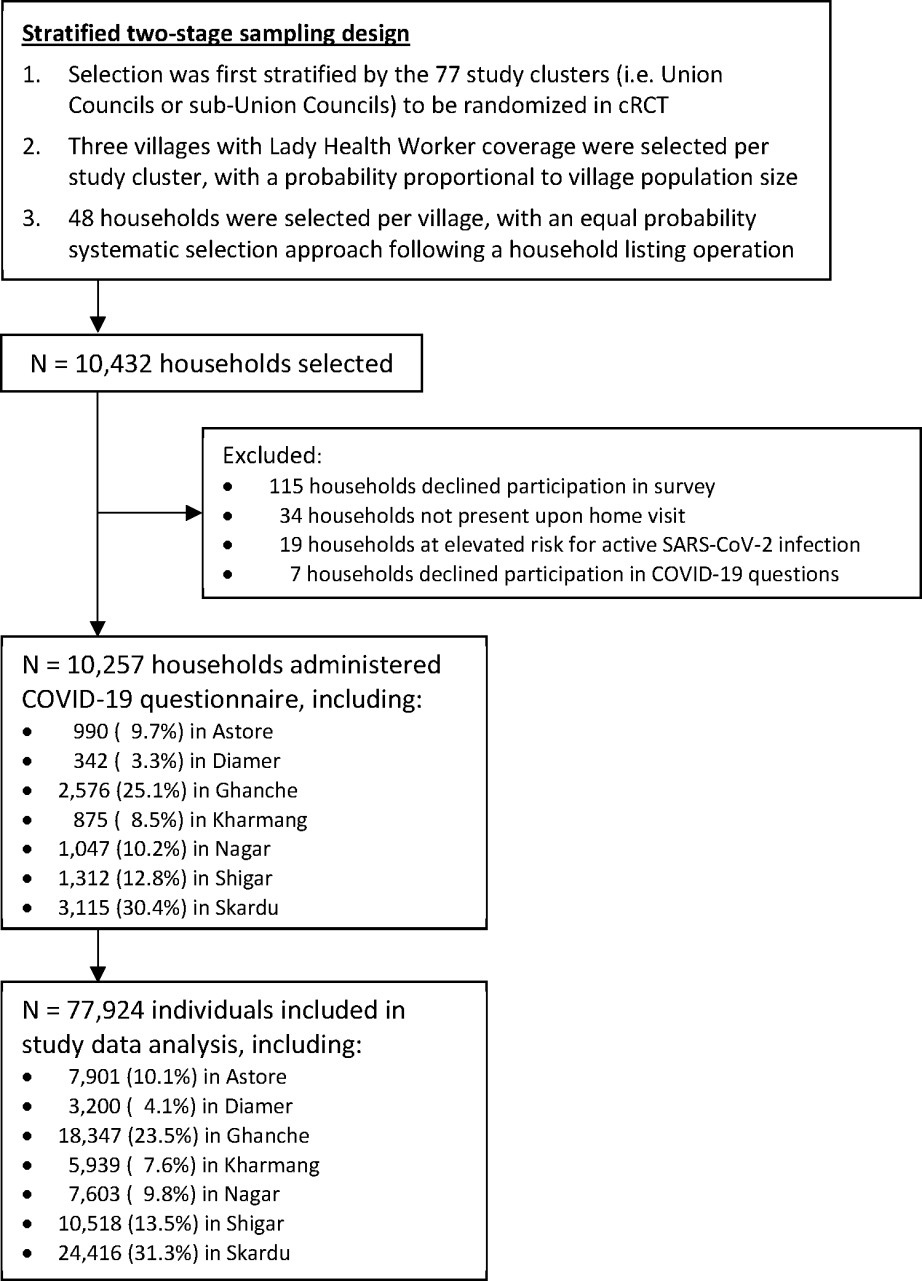
Sampling design and flow chart of households and individuals included in the COVID-19 survey in Gilgit-Baltistan, between June and August 2021. *For field team safety, households were not enrolled if they reported any household positive SARS-CoV-2 test, healthcare worker diagnosis of COVID-19 or were told to self-isolate in the past 14 days. This study, therefore, assesses prior SARS-CoV-2 infection. †These households participated in the primary series of questions regarding maternal and newborn outcomes, and subsequently declined to respond to questions regarding COVID-19. cRCT=Cluster randomized controlled trial.

From 14 June 2021 to 31 August 2021, 10 257 households participated in the household survey on COVID-19 (figure 1). Among all eligible households (n=10 432), 19 (0.2%) were excluded because they were at elevated risk for active SARS-CoV-2 infection at the time of interview. Individual histories were collected for 77 924 people, among whom the median age was 18 years (IQR 9–35 years), and 50.1% were female (online supplemental table S1, online supplemental figure S1). The median household size was seven individuals (IQR 5–9), and 66.3% of households had any Lady Health Worker coverage in the past 12 months.

### Epidemiology of COVID-19 testing and diagnoses

From all individuals, 314 (0.5%) reported a past confirmed/probable SARS-CoV-2 infection while 3263 (4.4%) had negative SARS-CoV-2 tests (table 1). Furthermore, 1080 (1.5%) had a COVID-19 positive contact, 605 (0.8%) had HCW diagnoses of ‘pneumonia/lung infection’, while most individuals (n=72 662, 92.8%) lacked any history of SARS-CoV-2 testing, diagnoses or close contacts. Among confirmed/probable infections, 167 had positive SARS-CoV-2 tests, 115 had HCW diagnoses of COVID-19 and 32 had HCW diagnoses of ‘pneumonia/lung infection’ plus COVID-19 positive contacts (online supplemental table S2).

**Table 1:** Presenting symptoms and outcomes among household members in Gilgit-Baltistan, by SARS-CoV-2 classification group, cumulatively from March 2020 to August 2021

Characteristic*	Known SARS-CoV-2 status			Unknown SARS-CoV-2 status		
	Confirmed or probable infection (N=314)	Negative (N=3263)	aPR (95% CI)†	With close contact (N=1080)	No close contact	
					With pneumonia/lung infection (N=605)	No pneumonia/ lung infection (N=72 662)
Age group (years), no. (%)						
<1	1 (0.3)	6 (0.1)	3.10 (0.66 to 14.63)	29 (2.5)	55 (9.7)	1870 (2.6)
1–4	15 (4.5)	59 (1.6)	**3.18(1.93to5.26)**	129 (12.2)	185 (33.4)	7840 (11.1)
5–9	17 (5.9)	111 (3.9)	**1.93(1.14to3.25)**	140 (12.9)	87 (15.2)	9985 (14.1)
10–14	14 (3.7)	215 (6.5)	0.79 (0.31 to 1.97)	150 (13.8)	58 (9.1)	9796 (13.5)
15–19	23 (6.7)	259 (7.4)	1.22 (0.76 to 1.96)	158 (14.7)	41 (6.4)	9198 (12.7)
20–29	62 (21.2)	649 (19.5)	1.45 (0.89 to 2.35)	190 (18.1)	29 (4.3)	11 927 (16.3)
30–39	45 (14.6)	625 (20.4)	Reference	102 (9.0)	28 (4.0)	7783 (10.6)
40–49	40 (12.7)	517 (15.8)	1.11 (0.73 to 1.70)	79 (8.1)	27 (3.9)	5470 (7.4)
50–59	39 (12.9)	349 (10.9)	1.57 (0.97 to 2.54)	37 (2.5)	28 (4.2)	3669 (4.9)
60–69	33 (11.6)	282 (8.4)	1.83 (0.92 to 3.63)	32 (3.2)	33 (4.4)	2943 (3.9)
≥70	25 (5.9)	191 (5.5)	1.45 (0.83 to 2.54)	34 (2.9)	34 (5.5)	2181 (2.9)
Age (years), median (IQR)	33 (21–52)	35 (22–49)	---	17 (8–30)	6 (2–23)	18 (8–33)
Sex, no. (%)						
Female	136 (42.8)	1323 (39.9)	Reference	556 (51.6)	310 (51.1)	36 631 (50.5)
Male	178 (57.2)	1940 (60.1)	0.88 (0.73 to 1.06)	524 (48.4)	295 (48.9)	36 031 (49.5)
Symptoms, no. (%)						
Fever	293 (93.5)	2523 (77.8)	**3.68(1.60to8.46)**	625 (57.4)	539 (92.2)	37 590 (51.8)
Cough	237 (79.4)	1486 (42.7)	**4.36(2.77to6.87)**	365 (35.2)	425 (72.7)	21 092 (27.7)
Headaches	213 (70.0)	1725 (52.3)	**2.17(1.33to3.55)**	332 (30.0)	287 (43.9)	20 638 (27.0)
Chills	213 (69.7)	1080 (31.6)	**4.26(2.33to7.76)**	307 (30.7)	268 (50.4)	14 655 (20.7)
Runny nose/congestion/sneezing	204 (67.1)	1445 (41.1)	**2.63(1.44to4.78)**	356 (34.0)	352 (61.6)	20 636 (26.9)
Sore throat	164 (56.2)	725 (21.2)	**3.93(2.13to7.25)**	198 (18.7)	203 (30.9)	9199 (11.6)
Difficulty breathing	143 (50.7)	370 (10.7)	**6.14(3.56to10.60)**	95 (9.4)	219 (37.5)	4151 (5.1)
Muscle pain	141 (48.0)	921 (26.2)	**2.54(1.63to3.96)**	134 (13.8)	149 (21.8)	7844 (10.2)
Loss of taste/smell	106 (38.0)	362 (9.9)	**4.21(2.67to6.64)**	81 (7.3)	76 (11.2)	3592 (4.2)
Vomiting	78 (27.3)	257 (6.8)	**3.62(2.33to5.62)**	90 (7.5)	202 (36.7)	7063 (9.0)
Diarrhoea	56 (18.0)	228 (6.4)	**2.40(1.42to4.03)**	106 (9.0)	176 (30.1)	7487 (9.7)
Number of symptoms, median (IQR)	6 (4–9)	3 (2–5)	**1.37(1.26to1.48)**	2 (0–4)	5 (3–6)	1 (0–3)
Zero symptoms, no. (%)	11 (4.3)	493 (15.5)	**0.28(0.09to0.87)**	385 (37.0)	24 (2.7)	30 823 (42.7)
Outcomes, no. (%)						
Sought medical treatment	276 (88.3)	2363 (69.4)	**3.01(1.32to6.85)**	518 (46.5)	515 (85.7)	30 588 (40.6)
Hospitalised	80 (26.6)	136 (4.0)	**5.49(3.68to8.20)**	14 (1.4)	166 (26.6)	352 (0.4)
Died	16 (5.2)	25 (0.7)	**4.63(3.06to7.00)**	6 (0.5)	19 (3.1)	50 (0.1)
Age at death (years), median (IQR)	68 (60–70)	70 (65–80)	---	64 (40–65)	18 (1–80)	60 (15–80)

* Summary statistics are presented as unweighted counts, weighted percentages and weighted continuous statistics.

† Prevalence ratios adjust for the individual’s age and sex. Specifically, age group and sex adjust for one another; all symptoms and outcomes adjust for both age group and sex. CIs not crossing the null value of 1 are bolded.

aPR, adjusted prevalence ratio; IQR, interquartile range.

Among confirmed/probable infections, fever (93.5%) and cough (79.4%) were the most common reported symptoms, while 4.3% reported no symptoms. Compared with individuals with negative tests, all symptoms and outcomes were associated with increased prevalence of confirmed/probable infection, adjusting for age and sex. Difficulty breathing (aPR 6.14, 95% CI 3.56 to 10.60) and hospitalisation (aPR 5.49, 95% CI 3.68 to 8.20) were most strongly associated with a history of confirmed or probable infection among all individuals. Age-specific symptoms and outcomes are presented in online supplemental tables S3–S7. Gastrointestinal symptoms including vomiting (77.2%) and diarrhoea (60.7%) were more common among children <5 years, relative to all respondents (27.3% and 18.0%; online supplemental table S3). In addition, 80 individuals (26.6%) were hospitalised while 16 (5.2%) died. The median age of death was 68 years (IQR 60–70). Age ≥70 years was associated with increased risk of hospitalisation or death (PR 2.40, 95% CI 1.16 to 4.98) vs ages 30–39 years (online supplemental table S8), and 18.2% of those who were hospitalised or died had chronic health conditions (online supplemental table S9).

Individuals with HCW diagnoses of ‘pneumonia/lung infection’ demonstrated a similar symptom profile as those with confirmed/probable SARS-CoV-2 infection (eg, 92.2% vs 93.5% with fever, 72.7% vs 79.4% with cough and an identical 26.6% hospitalised), despite being younger on average (median age 6 years (IQR 2–23) vs 33 years (IQR 21–52)). Individuals with COVID-19 positive contacts and those without either positive contacts or HCW diagnoses experienced a lower burden of symptoms (eg, 57.4% and 51.8% with fever, 35.2% and 27.7% with cough, 1.4% and 0.4% hospitalised), and their age reflected the overall population distribution (median age 17 and 18 years, respectively).

SARS-CoV-2 testing rates differed by age and sex (table 2). From March 2020 to June/August 2021, cumulative testing rates per 1000 population were highest for ages 50–59 years (102.7 tests per 1000) and lowest for ages <1 year (2.5 tests per 1000) and 1–4 years (7.4 tests per 1000). Meanwhile, males were more likely to be tested than females (age-adjusted PR 1.51, 95% CI 1.40 to 1.63), with cumulative testing rates of 57.8 tests per 1000 males and 38.0 tests per 1000 females. As a result, individuals with confirmed/probable infection were typically older (median age 33 years) and male (57.2% vs 42.8% female) than the overall population, though these differences were less pronounced among those with probable COVID-19. Despite some regional differences, the total rate of SARS-CoV-2 testing identified in this study (47.9 tests per 1000 people; 95% CI 42.0 to 53.9) was similar to rates reported by the Gilgit-Baltistan Department of Health (42.3 tests per 1000 people; online supplemental table S10).

**Table 2:** Age and sex of individuals tested versus not tested for SARS-CoV-2 among household members in Gilgit-Baltistan between March 2020 and August 2021

Characteristic*	Tested for SARS-CoV-2		aPR (95% CI)†	Tests per 1000 population (95% CI)	Test positivity, % (95% CI)
	Yes (N=3546)	No (N=74 378)			
Age group (years), no. (%)					
<1	7 (0.1)	1954 (2.7)	**0.03(0.01to0.07)**	2.5 (0.5 to 4.6)	0.0 (0.0 to 0.0)
1–4	68 (1.7)	8160 (11.3)	**0.08(0.06to0.12)**	7.4 (4.9 to 10.0)	1.1 (0.8 to 1.4)
5–9	129 (4.2)	10 211 (14.1)	**0.17(0.12to0.23)**	14.9 (10.5 to 19.2)	1.6 (0.2 to 12.7)
10–14	233 (6.6)	10 000 (13.4)	**0.28(0.22to0.35)**	24.0 (18.8 to 29.2)	0.7 (0.1 to 3.2)
15–19	283 (7.5)	9396 (12.7)	**0.33(0.28to0.41)**	29.0 (22.9 to 35.0)	3.6 (1.6 to 7.8)
20–29	700 (19.3)	12 157 (16.2)	**0.66(0.58to0.75)**	56.6 (46.1 to 67.1)	5.5 (3.0 to 9.8)
30–39	664 (19.8)	7919 (10.5)	Reference	86.5 (73.3 to 99.8)	4.4 (2.0 to 9.0)
40–49	552 (15.4)	5581 (7.4)	1.09 (0.95 to 1.25)	95.3 (79.8 to 110.7)	3.8 (2.0 to 7.1)
50–59	386 (11.1)	3736 (4.9)	**1.18(1.04to1.35)**	102.7 (87.8 to 117.5)	9.7 (6.5 to 14.5)
60–69	312 (8.7)	3011 (3.9)	1.13 (0.94 to 1.35)	100.0 (83.8 to 116.2)	12.0 (5.4 to 24.7)
≥70	212 (5.6)	2253 (3.0)	0.95 (0.77 to 1.17)	86.3 (72.4 to 100.2)	7.2 (4.3 to 11.9)
Sex, no. (%)					
Female	1433 (39.7)	37 523 (50.6)	Reference	38.0 (33.1 to 43.0)	5.0 (3.2 to 7.7)
Male	2113 (60.3)	36 855 (49.4)	**1.51(1.40to1.63)**	57.8 (50.5 to 65.2)	5.8 (3.9 to 8.6)

* Summary statistics are presented as unweighted counts and weighted percentages or rates.

† Adjusted prevalence ratios were generated from one model, including both age category and sex. CIs not crossing the null value of 1 are bolded.

aPR, adjusted prevalence ratio.

### Estimation of unconfirmed COVID-19 cases

**Figure 2:**
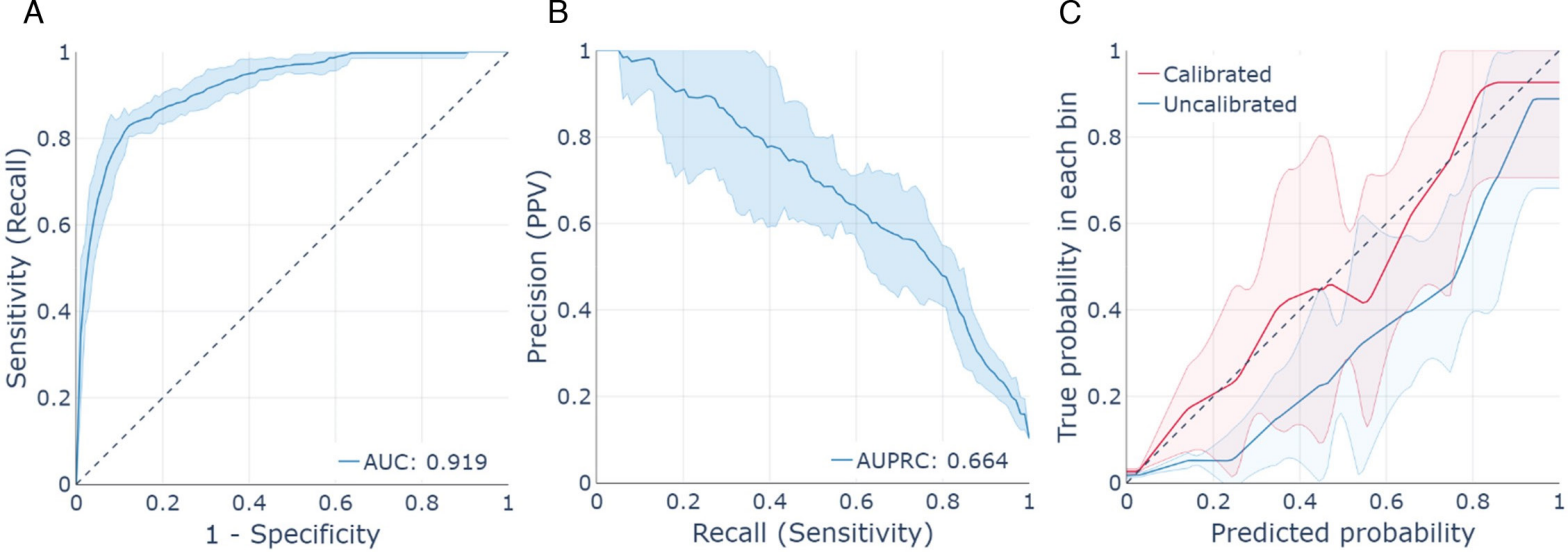
Validation figures for the XGBoost model differentiating confirmed/probable SARS-CoV-2 infections (n=314) from test-negative individuals (n=3263), including (A) receiver operating characteristic curve, (B) precision-recall curve and (C) calibration curve. AUC, Area under the curve; AUPRC, area under the precision-recall curve; PPV=Positive predictive value.

Machine learning model training/selection and testing/validation was conducted among 3577 participants with known SARS-CoV-2 history, including 314 confirmed/probable infections vs 3263 with negative tests. Hyperparameter values and validation statistics for all trained models are described in online supplemental figures S2–S7, online supplemental tables S11–S13. The final XGBoost model had an auROC of 0.919 (95% CI 0.904 to 0.930), auPRC of 0.664 (95% CI 0.578 to 0.729), sensitivity of 0.813 (95% CI 0.747 to 0.853) and specificity of 0.877 (95% CI 0.849 to 0.903) (figure 2, online supplemental table S12). The most important features of the XGBoost model were individual reports of difficulty breathing, cough and the Z-score normalised rate of village-level confirmed/probable infections (online supplemental figure S8).

Applying the final model to individuals with unknown SARS-CoV-2 history, the model predicted 1264 positive SARS-CoV-2 infections (95% CI 907 to 1740). Considering both the XGBoost predictions and probable COVID-19 cases (ie, HCW diagnoses of COVID-19 or ‘pneumonia/lung infection’ with COVID-19-positive contact), total estimated cases were 8.4 times higher than the number of individuals with positive SARS-CoV-2 tests (table 3, online supplemental table S14). If possible COVID-19 was also included (ie, those with COVID-19 positive contacts or HCW diagnoses of ‘pneumonia/lung infection’), total estimated cases were 17.4 times higher than the number of positive SARS-CoV-2 tests. There were substantially more unconfirmed cases among children, with 211.0 probable cases (480.0 probable or possible cases) per confirmed case for ages 1–4 years and 79.5 probable cases (185.0 probable or possible cases) per confirmed case for ages 5–9, compared with 2.8 probable cases (4.7 probable or possible cases) per confirmed case for ages 60–69 years. There were also twice as many unconfirmed cases among females (1 positive test: 12.5 estimated probable cases) compared with males (1 positive test: 6.2 estimated probable cases).

**Table 3:** Number of confirmed, estimated probable and estimated possible COVID-19 cases in Gilgit-Baltistan, cumulatively from March 2020 to August 2021

Characteristic	Confirmed SARS-CoV-2, no. cases	Probable COVID-19*, no. cases (95% CI)	Possible COVID-19†, no. cases (95% CI)	Ratio of confirmed to estimated cases	
				Confirmed : probable	Confirmed : probable+possible
Overall	167	1411 (1054 to 1887)	1491 (1423 to 1546)	1:8.4	1:17.4
Age group (years)					
<1	0	50 (20 to 101)	71 (62 to 80)	---	---
1–4	1	211 (107 to 419)	269 (234 to 293)	1:211.0	1:480.0
5–9	2	159 (77 to 283)	211 (196 to 223)	1:79.5	1:185.0
10–14	2	127 (63 to 209)	195 (186 to 204)	1:63.5	1:161.0
15–19	9	151 (97 to 237)	177 (168 to 184)	1:16.8	1:36.4
20–29	33	224 (143 to 344)	196 (184 to 205)	1:6.8	1:12.7
30–39	25	132 (84 to 184)	115 (110 to 120)	1:5.3	1:9.9
40–49	20	107 (74 to 148)	95 (89 to 99)	1:5.4	1:10.1
50–59	30	83 (54 to 117)	58 (54 to 62)	1:2.8	1:4.7
60–69	28	92 (61 to 130)	50 (45 to 55)	1:3.3	1:5.1
≥70	17	65 (26 to 110)	56 (47 to 63)	1:3.8	1:7.1
Sex					
Female	60	744 (548 to 1009)	767 (735 to 794)	1 : 12.5	1 : 25.2
Male	107	667 (475 to 925)	724 (685 to 759)	1 : 6.2	1 : 13.0
District					
Astore	6	45 (18 to 85)	126 (116 to 133)	1 : 7.5	1 : 28.5
Diamer	20	353 (209 to 517)	100 (80 to 117)	1 : 17.7	1 : 22.7
Ghanche	24	230 (80 to 444)	209 (195 to 220)	1 : 9.6	1 : 18.3
Kharmang	3	3 (0 to 11)	52 (51 to 52)	1 : 1.0	1 : 18.3
Nagar	27	379 (262 to 492)	280 (257 to 307)	1 : 14.0	1 : 24.4
Shigar	14	30 (8 to 74)	164 (159 to 167)	1 : 2.1	1 : 13.9
Skardu	73	371 (241 to 574)	560 (532 to 579)	1 : 5.1	1 : 12.8

* Individuals with healthcare worker diagnoses of COVID-19, healthcare worker diagnoses of pneumonia/lung infection with positive close contact or positive machine learning predictions.

† Individuals with COVID-19-positive close contacts (and without pneumonia/lung infection) and HCW diagnoses of pneumonia/lung infection (and without positive close contacts). Cases who were predicted as positive COVID-19 in the machine learning analysis are not double counted (ie, only included in the ‘probable’ column).

HCW, healthcare worker.

## Discussion

We collected information regarding SARS-CoV-2 symptoms, diagnoses and outcomes among nearly 78 000 people in Gilgit-Baltistan, a remote and mountainous region in northern Pakistan. We estimate the true number of SARS-CoV-2 infections was at least 8–17 times greater than cases detected by clinical and public health testing infrastructure between March 2020 and August 2021, a period when ancestral and Alpha lineages were predominant in Pakistan.^30^ Our XGBoost predictive model demonstrated high discrimination between confirmed/probable infections and individuals with negative SARS-CoV-2 tests (auROC=0.919). Consistent with prior studies, respiratory symptoms including cough and difficulty breathing, as well as variables denoting proximity to sick contacts, were most strongly associated with SARS-CoV-2 infection.^11 12^ However, the use of a primarily symptom-based model likely resulted in misclassification of asymptomatic and mildly symptomatic infections. For instance, prior seroprevalence studies in low-middle-income settings have estimated a greater total underdetection of SARS-CoV-2 infection, including in India (1 detected case per 26–32 infections between August and September 2020),^31^ Indonesia (1 detected case per 52 infections between October and December 2020)^32^ and Zambia (1 detected case per 92 infections in July 2020).^16^ As such, the ratio of underdetection estimated in this study is likely an underestimate of the true ratio. Nevertheless, we demonstrate that machine learning may be used to highlight SARS-CoV-2 underdiagnosis in settings with sparse healthcare infrastructure and minimal capacity for SARS-CoV-2 molecular and serologic testing. While seroprevalence studies may estimate the burden of infection more completely, machine learning approaches are likely to be more cost-effective and feasible to implement, while still being based on primary data.

Our study accounted for multiple avenues of SARS-CoV-2 underdetection and underreporting, including HCW diagnoses in the absence of testing and infected individuals not interfacing with healthcare services. We found that for each person who tested positive, one additional individual received a HCW diagnosis of COVID-19 in the absence of testing, similar to a cross-sectional mobile survey among 5000 adults across Pakistan.^33^ HCW diagnoses of syndromic ‘pneumonia/lung infection’ were twice as frequent as COVID-19 diagnoses, with a similar symptom profile compared with confirmed/probable infections despite a younger age distribution. While not completely eliminated as in many high-income settings, Pakistan experienced declines in influenza, respiratory syncytial virus and non-SARS-CoV-2 upper respiratory tract infections in 2020–2021, suggesting at least some of these HCW diagnoses were attributable to SARS-CoV-2.^34 35^ Moreover, in a Pakistani study conducted in October–November 2020, seroprevalence of SARS-CoV-2 antibodies in Gilgit district was estimated as 17%.^36^ Notably, underdetection of COVID-19-related death was less pronounced than for SARS-CoV-2 infection. While models predicting COVID-19 mortality were not developed given a relatively low number of deaths, 75 deaths were reported among untested individuals (including 6 with positive contacts and 19 with pneumonia/lung infection). When compared with deaths among individuals with confirmed/probable infection (n=16), the true number of COVID-19-related deaths would be at most 4.6 times greater than confirmed or probable COVID-19-related deaths.

There were differences in SARS-CoV-2 testing rates by age and sex, whereby males were 1.5 times more likely to be tested than females, and individuals aged 50–59 years were 13.9 times more likely to be tested than those aged 1–4 years. In Gilgit-Baltistan, SARS-CoV-2 testing was mostly conducted among hospitalised individuals or those crossing international or regional borders.^8 37^ Older age and male sex are both established risk factors for severe COVID-19, which may partially explain a male predominance in testing rates.^38^ Adult males in Gilgit-Baltistan are also most likely to engage in paid employment outside the home, sometimes requiring seasonal travel across regional borders. Conversely, women in Gilgit-Baltistan face consistent barriers to accessing healthcare services including a lack of autonomy for both health and travel-related spending, stigmas associated with unaccompanied travel, and low rates of female health providers in facility settings.^39^ These barriers may explain the underdetection of SARS-CoV-2 infections among females (1 positive test: 12–25 estimated probable cases), and separately children (eg, 1 positive test: 211–480 estimated probable cases among ages 1–4 years). The low rate of testing among these populations also impedes detection during the pregnancy and neonatal periods, both associated with increased risk of COVID-19-related complications.^40^

This study is subject to several limitations. First, false positive and negative results may have occurred among those tested for SARS-CoV-2, those receiving HCW COVID-19 diagnoses and in the machine learning analysis. Second, all data were self-reported and collected using a recall period of 15–17 months and may be susceptible to recall bias. Third, the exclusion of households screening positive for active infection may contribute to further underestimation; thus, our results should be interpreted as describing prior (and not current) infections. Fourth, the model was developed disproportionately among adults and also predicted many false positive results, perhaps owing to the non-specificity of COVID-19-related symptoms. However, despite some age differences in clinical presentations, the model still predicts a much larger ratio of confirmed to estimated cases among children and youth than in adults. Fifth, those with negative SARS-CoV-2 tests presented with a systematically higher burden of symptoms than those with unknown SARS-CoV-2 histories, and it is unclear how this may have affected generalisability when predicting past SARS-CoV-2 diagnoses. Moreover, given changes in population-level immunity to SARS-CoV-2, differences in current circulating lineages, as well as the resurgence of non-SARS-CoV-2 respiratory viruses, the predictive model may not be generalisable to future studies. Sixth, we did not collect temporal data and therefore cannot comment on the timing of estimated probable cases in this study. Finally, household ownership of thermometers in Gilgit-Baltistan is uncommon, and rates of fever likely include a substantial degree of perceived fever measured through touch.

## Conclusions

In summary, we present comprehensive information regarding the burden of confirmed and unconfirmed COVID-19 in Gilgit-Baltistan and demonstrate that the large majority of SARS-CoV-2 infections went undetected, particularly among women and young children. As rates of SARS-CoV-2 testing decline globally, our approach of combining primary data sources of unconfirmed cases with machine learning techniques may be used to inform the ongoing missed burden of COVID-19. Our findings may be used to inform healthcare and laboratory resource allocation decision-making by policy-makers in Pakistan, and may be useful in other global contexts and future disease outbreaks. Furthermore, this methodology may have the potential to be extended to other infections and disease entities, and future research is recommended.

## Supplementary material

10.1136/bmjph-2024-001255 Supplementary file 1

## Data Availability

Data are available on reasonable request.
