# Supplementary material for: Estimation of unconfirmed COVID-19 cases from a cross-sectional survey of >10 000 households and a symptom-based machine learning model in Gilgit-Baltistan, Pakistan
Source: BMJ Public Health. 2025 Apr 28;3(1):e001255. doi: 10.1136/bmjph-2024-001255 (PMC12039044; doi:10.1136/bmjph-2024-001255)
Supplement: Supplementary file 1 [file bmjph-3-1-s001.docx]

**Supplementary Materials:** Estimation of unconfirmed COVID-19 cases from a cross-sectional survey of >10,000 households and a symptom-based machine learning model in Gilgit-Baltistan, Pakistan

**Table of Contents**

[Supplementary Figures and Tables 2](#_Toc184984481)

[Table S1. Demographic characteristics of households enrolled at baseline. 2](#_Toc184984482)

[Table S2. Age and sex of individuals with confirmed or probable COVID-19. 4](#_Toc184984483)

[Table S3. Presenting symptoms and outcomes by SARS-CoV-2 classification group, among children <5 years old. 5](#_Toc184984484)

[Table S4. Presenting symptoms and outcomes by SARS-CoV-2 classification group, among children 5–9 years old. 6](#_Toc184984485)

[Table S5. Presenting symptoms and outcomes by SARS-CoV-2 classification group, among children and youth 10–19 years old. 7](#_Toc184984486)

[Table S6. Presenting symptoms and outcomes by SARS-CoV-2 classification group, among adults 20–49 years old. 8](#_Toc184984487)

[Table S7. Presenting symptoms and outcomes by SARS-CoV-2 classification group, among adults ≥50 years old. 9](#_Toc184984488)

[Table S8. Characteristics of participants with confirmed or probable COVID-19, by disease severity. 10](#_Toc184984489)

[Table S9. Chronic conditions amongst hospitalized or deceased individuals, by SARS-CoV-2 classification group. 11](#_Toc184984490)

[Table S10. Comparison of study indicators versus GB Department of Health statistics. 12](#_Toc184984491)

[Appendix 1: Calculation of SARS-CoV-2 testing rates 13](#_Toc184984492)

[Appendix 2: COVID-19 study data collection form 14](#_Toc184984493)

[Appendix 3: Machine learning model selection and validation 16](#_Toc184984494)

[Figure S2. Workflow diagram for machine learning analysis. 20](#_Toc184984495)

[Table S11. Hyperparameter values included in the 10-fold grid search cross validation. 21](#_Toc184984496)

[Table S12. Validation statistics and bootstrapped 95% confidence intervals for each trained ML algorithm. 22](#_Toc184984497)

[Figure S3. Receiver operating characteristic curve (ROC; left), precision-recall curve (PRC; centre), and calibration curve for logistic regression model. 23](#_Toc184984498)

[Figure S4. Receiver operating characteristic curve (ROC; left), precision-recall curve (PRC; centre), and calibration curve for support vector machine model. 23](#_Toc184984499)

[Figure S5. Receiver operating characteristic curve (ROC; left), precision-recall curve (PRC; centre), and calibration curve for random forest model. 24](#_Toc184984500)

[Figure S6. Receiver operating characteristic curve (ROC; left), precision-recall curve (PRC; centre), and calibration curve for voting classifier ensemble model. 24](#_Toc184984501)

[Figure S7. Confusion matrices for the five machine learning algorithms. 25](#_Toc184984502)

[Table S13. Validation statistics and bootstrapped 95% confidence intervals for the XGBoost model, at varying thresholds of sensitivity (recall) and specificity. 26](#_Toc184984503)

[Figure S8. Feature importance coefficients and 95% confidence intervals of the selected XGBoost model. 27](#_Toc184984504)

[Table S14. Breakdown of confirmed, probable, and possible COVID-19 cases. 28](#_Toc184984505)

# **Supplementary Figures and Tables**

| **Table S1. Demographic characteristics of households enrolled at baseline.** | | |
| --- | --- | --- |
| **Characteristic*** | | **Distribution** |
| **Household characteristics** | | |
| **Number of enrolled households, no.** | | 10257 |
| **District, no. (%)** | |  |
|  | Astore | 990 (9.7) |
|  | Diamer | 342 (3.3) |
|  | Ghanche | 2576 (25.1) |
|  | Kharmang | 875 (8.5) |
|  | Nagar | 1047 (10.2) |
|  | Shigar | 1312 (12.8) |
|  | Skardu | 3115 (30.4) |
| **Household size, median (IQR)** | | 7 (5–9) |
|  | (min, max) | (1, 40) |
| **LHW home visits (past year), median (IQR)** | | 4 (0–6) |
|  | (min, max) | (0, 28) |
|  | Households with zero visits, no. (%)† | 3274 (33.7) |
| **Individual characteristics** | | |
| **Number of individuals, no.** | | 77924 |
| **Age (years), median (IQR)** | | 18 (9–35) |
| **Age group (years), no. (%)** | |  |
|  | <1 | 1961 (2.6) |
|  | 1 to 4 | 8228 (10.8) |
|  | 5 to 9 | 10340 (13.4) |
|  | 10 to 14 | 10233 (13.1) |
|  | 15 to 19 | 9679 (12.4) |
|  | 20 to 29 | 12857 (16.3) |
|  | 30 to 39 | 8583 (11.0) |
|  | 40 to 49 | 6133 (7.8) |
|  | 50 to 59 | 4122 (5.2) |
|  | 60 to 69 | 3323 (4.2) |
|  | ≥70 | 2465 (3.1) |
| **Sex, no. (%)** | |  |
|  | Female | 38956 (50.1) |
|  | Male | 38968 (49.9) |
| *With the exception of study district, summary statistics are presented as unweighted counts, weighted percentages, and weighted continuous statistics. †Data missing for 188 households. | | |


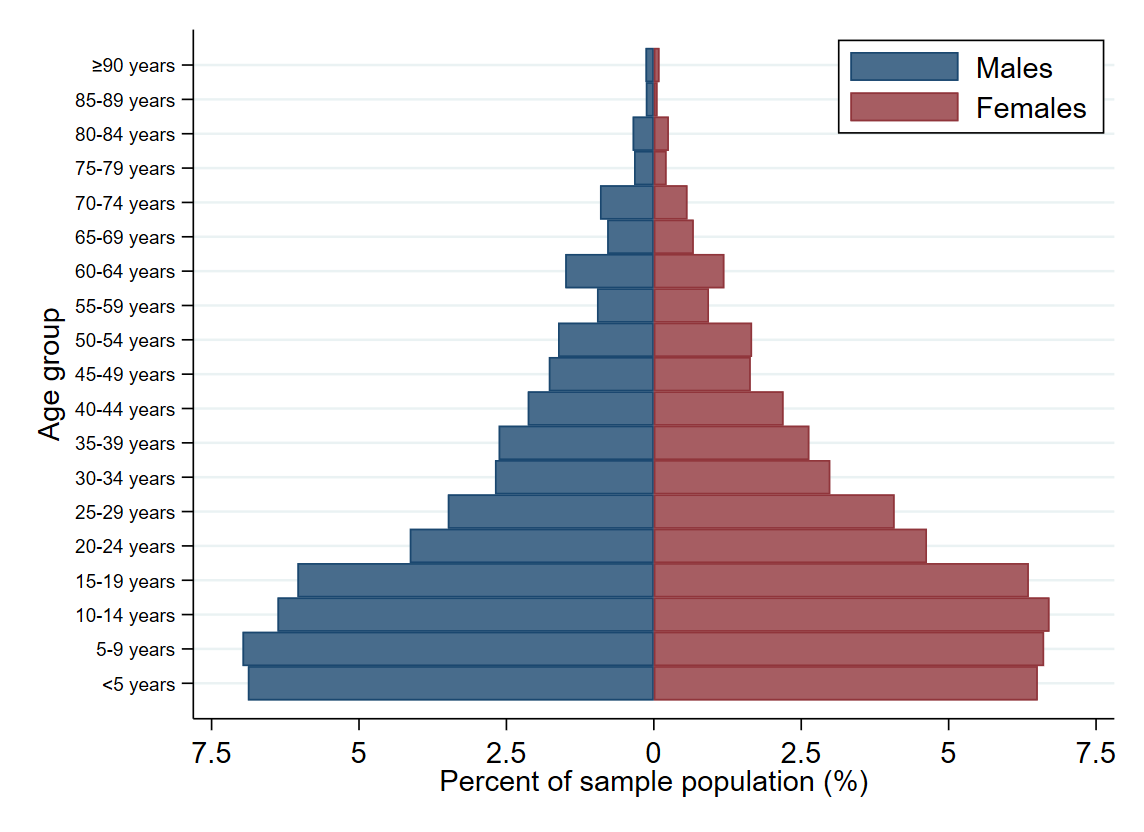
 Figure S1. Population pyramid showing distribution of the sample population by age and sex.

| **Table S2. Age and sex of individuals with confirmed or probable COVID-19.** | | | | |
| --- | --- | --- | --- | --- |
| **Characteristic*** | | **Confirmed COVID-19 (N = 167)** | **Probable COVID-19** | |
|  |  |  | **HCW diagnosis of COVID-19 (N = 115)** | **HCW diagnosis of ‘pneumonia/lung infection’ AND COVID-19 positive contact (2) (N = 32)** |
| **Age (years), median (IQR)** | | 45 (28–60) | 25 (16–41) | 20 (6–40) |
| **Age group (years), no. (%)** | |  |  |  |
|  | <1 | 0 (0.0) | 1 (0.8) | 0 (0.0) |
|  | 1 to 4 | 1 (0.3) | 7 (6.2) | 7 (20.0) |
|  | 5 to 9 | 2 (1.0) | 8 (7.8) | 7 (24.0) |
|  | 10 to 14 | 2 (0.8) | 10 (7.4) | 2 (4.3) |
|  | 15 to 19 | 9 (4.7) | 13 (10.6) | 1 (1.0) |
|  | 20 to 29 | 33 (19.4) | 27 (26.4) | 2 (7.9) |
|  | 30 to 39 | 25 (15.9) | 16 (13.0) | 4 (14.2) |
|  | 40 to 49 | 20 (10.9) | 17 (14.5) | 3 (15.2) |
|  | 50 to 59 | 30 (20.1) | 7 (5.3) | 2 (5.5) |
|  | 60 to 69 | 28 (19.3) | 4 (4.3) | 1 (0.9) |
|  | ≥70 | 17 (7.5) | 5 (3.6) | 3 (7.1) |
| **Sex, no. (%)** | |  |  |  |
|  | Female | 60 (36.1) | 57 (47.9) | 19 (58.1) |
|  | Male | 107 (63.9) | 58 (52.1) | 13 (41.9) |
| *Summary statistics are presented as unweighted counts, and weighted percentages or rates. | | | | |

| **Table S3. Presenting symptoms and outcomes by SARS-CoV-2 classification group, among children <5 years old.** | | | | | | | |
| --- | --- | --- | --- | --- | --- | --- | --- |
| **Characteristic*** | | **Known SARS-CoV-2 status** | |  | **Unknown SARS-CoV-2 status** | | |
|  |  | **Confirmed or probable infection (N = 16)** | **Negative (N = 65)** |  | **With close contact (N = 158)** | **No close contact** | |
|  |  |  |  |  |  | **With pneumonia/ lung infection (N = 240)** | **No pneumonia/ lung infection (N = 9710)** |
| **Sex, no. (%)** | |  |  |  |  |  |  |
|  | Female | 11 (71.8) | 23 (34.5) |  | 76 (51.6) | 99 (38.3) | 4762 (48.9) |
|  | Male | 5 (28.2) | 42 (65.5) |  | 82 (48.4) | 141 (61.7) | 4948 (51.1) |
| **Symptoms, no. (%)** | |  |  |  |  |  |  |
|  | Fever | 15 (96.0) | 53 (77.5) |  | 93 (62.5) | 223 (96.3) | 5184 (55.0) |
|  | Cough | 14 (87.7) | 27 (40.6) |  | 52 (34.5) | 168 (72.8) | 2690 (27.5) |
|  | Chills | 12 (71.8) | 15 (20.7) |  | 43 (27.9) | 101 (50.5) | 1756 (20.2) |
|  | Runny nose/congestion/sneezing | 12 (71.8) | 28 (41.6) |  | 54 (34.3) | 153 (67.6) | 2668 (27.7) |
|  | Vomiting | 12 (77.2) | 15 (24.5) |  | 31 (16.4) | 123 (55.8) | 2362 (23.2) |
|  | Diarrhea | 11 (60.7) | 17 (34.1) |  | 48 (28.5) | 108 (44.8) | 2540 (25.5) |
|  | Headaches | 9 (50.4) | 15 (21.3) |  | 23 (16.0) | 76 (28.0) | 1519 (12.8) |
|  | Sore throat | 8 (51.5) | 10 (12.8) |  | 20 (15.4) | 65 (23.4) | 932 (9.2) |
|  | Difficulty breathing | 7 (40.3) | 7 (9.1) |  | 7 (4.4) | 68 (28.6) | 417 (4.0) |
|  | Muscle pain | 4 (24.7) | 7 (10.3) |  | 2 (0.9) | 15 (4.3) | 222 (2.1) |
|  | Loss of taste/smell | 3 (22.9) | 3 (4.6) |  | 5 (3.5) | 10 (3.6) | 155 (1.5) |
| **Number of symptoms, median (IQR)** | | 6 (4–10) | 3 (2–4) |  | 2 (0–4) | 5 (3–6) | 1 (0–4) |
|  | Zero symptoms, no. (%) | 0 (0.0) | 9 (14.8) |  | 56 (33.1) | 4 (0.9) | 4066 (40.9) |
| **Outcomes, no. (%)** | |  |  |  |  |  |  |
|  | Sought medical treatment | 15 (93.9) | 53 (81.8) |  | 81 (52.9) | 209 (86.6) | 4302 (43.6) |
|  | Hospitalized | 5 (29.3) | 1 (1.1) |  | 1 (1.5) | 88 (34.6) | 78 (0.8) |
|  | Died | 0 (0.0) | 0 (0.0) |  | 0 (0.0) | 6 (2.4) | 12 (<0.1) |
| *Summary statistics are presented as unweighted counts, weighted percentages, and weighted continuous statistics. | | | | | | | |

| **Table S4. Presenting symptoms and outcomes by SARS-CoV-2 classification group, among children 5–9 years old.** | | | | | | | |
| --- | --- | --- | --- | --- | --- | --- | --- |
| **Characteristic*** | | **Known SARS-CoV-2 status** | |  | **Unknown SARS-CoV-2 status** | | |
|  |  | **Confirmed or probable infection (N = 17)** | **Negative (N = 111)** |  | **With close contact (N = 140)** | **No close contact** | |
|  |  |  |  |  |  | **With pneumonia/ lung infection (N = 87)** | **No pneumonia/ lung infection (N = 9985)** |
| **Sex, no. (%)** | |  |  |  |  |  |  |
|  | Female | 5 (33.6) | 62 (48.7) |  | 58 (41.0) | 46 (52.5) | 4819 (48.8) |
|  | Male | 12 (66.4) | 49 (51.3) |  | 82 (59.0) | 41 (47.5) | 5166 (51.2) |
| **Symptoms, no. (%)** | |  |  |  |  |  |  |
|  | Fever | 15 (89.4) | 82 (78.3) |  | 68 (50.4) | 77 (91.3) | 5046 (51.5) |
|  | Cough | 13 (78.6) | 49 (47.4) |  | 46 (37.7) | 63 (70.9) | 2723 (25.7) |
|  | Chills | 11 (65.3) | 20 (12.8) |  | 39 (32.3) | 35 (49.5) | 1744 (18.9) |
|  | Runny nose/congestion/sneezing | 9 (53.0) | 46 (31.7) |  | 38 (26.3) | 45 (57.2) | 2659 (25.2) |
|  | Headaches | 8 (49.2) | 32 (20.4) |  | 30 (16.5) | 41 (37.9) | 1961 (17.2) |
|  | Vomiting | 7 (47.1) | 14 (9.3) |  | 15 (9.0) | 26 (31.1) | 1313 (11.4) |
|  | Sore throat | 6 (38.9) | 15 (11.3) |  | 20 (11.7) | 27 (28.6) | 1038 (9.5) |
|  | Difficulty breathing | 6 (32.3) | 4 (4.7) |  | 6 (2.7) | 27 (31.6) | 375 (3.2) |
|  | Diarrhea | 5 (29.3) | 21 (13.7) |  | 18 (9.3) | 23 (25.7) | 1472 (13.2) |
|  | Muscle pain | 3 (19.1) | 5 (3.7) |  | 9 (6.1) | 11 (11.3) | 313 (2.8) |
|  | Loss of taste/smell | 1 (10.4) | 7 (3.3) |  | 6 (4.1) | 4 (3.0) | 229 (1.9) |
| **Number of symptoms, median (IQR)** | | 5 (3–7) | 2 (1–3) |  | 1 (0–4) | 5 (3–6) | 1 (0–3) |
|  | Zero symptoms, no. (%) | 1 (7.2) | 26 (19.2) |  | 64 (45.6) | 4 (3.0) | 4503 (44.8) |
| **Outcomes, no. (%)** | |  |  |  |  |  |  |
|  | Sought medical treatment | 13 (77.7) | 68 (49.0) |  | 55 (40.6) | 70 (82.7) | 3997 (38.5) |
|  | Hospitalized | 3 (16.6) | 5 (4.8) |  | 0 (0.0) | 21 (22.6) | 35 (3.1) |
|  | Died | 1 (5.6) | 0 (0.0) |  | 0 (0.0) | 1 (0.8) | 1 (<0.1) |
| *Summary statistics are presented as unweighted counts, weighted percentages, and weighted continuous statistics. | | | | | | | |

| **Table S5. Presenting symptoms and outcomes by SARS-CoV-2 classification group, among children and youth 10–19 years old.** | | | | | | | |
| --- | --- | --- | --- | --- | --- | --- | --- |
| **Characteristic*** | | **Known SARS-CoV-2 status** | |  | **Unknown SARS-CoV-2 status** | | |
|  |  | **Confirmed or probable infection (N = 37)** | **Negative (N = 474)** |  | **With close contact (N = 308)** | **No close contact** | |
|  |  |  |  |  |  | **With pneumonia/ lung infection (N = 99)** | **No pneumonia/ lung infection (N = 18994)** |
| **Sex, no. (%)** | |  |  |  |  |  |  |
|  | Female | 19 (45.8) | 247 (53.2) |  | 155 (49.9) | 49 (58.4) | 9660 (51.2) |
|  | Male | 18 (54.2) | 227 (46.8) |  | 153 (50.1) | 50 (41.6) | 9334 (48.8) |
| **Symptoms, no. (%)** | |  |  |  |  |  |  |
|  | Fever | 33 (90.2) | 306 (62.8) |  | 176 (57.4) | 84 (88.5) | 9219 (48.2) |
|  | Chills | 26 (78.9) | 102 (19.2) |  | 84 (32.0) | 34 (40.3) | 3286 (17.9) |
|  | Headaches | 21 (61.9) | 166 (30.6) |  | 97 (31.2) | 45 (48.1) | 4897 (24.0) |
|  | Cough | 20 (60.1) | 167 (34.3) |  | 96 (35.4) | 65 (75.9) | 5028 (24.9) |
|  | Runny nose/congestion/sneezing | 20 (57.6) | 185 (33.7) |  | 100 (36.6) | 52 (59.8) | 4993 (24.7) |
|  | Sore throat | 14 (45.7) | 84 (15.8) |  | 57 (19.7) | 30 (31.0) | 2231 (10.8) |
|  | Vomiting | 8 (26.1) | 31 (4.4) |  | 16 (5.8) | 18 (17.4) | 1264 (6.0) |
|  | Difficulty breathing | 9 (26.0) | 23 (3.7) |  | 26 (10.9) | 31 (41.6) | 836 (4.0) |
|  | Loss of taste/smell | 6 (22.0) | 28 (5.5) |  | 26 (9.0) | 12 (15.3) | 658 (3.0) |
|  | Muscle pain | 5 (16.2) | 46 (9.0) |  | 31 (13.1) | 18 (17.3) | 1013 (5.2) |
|  | Diarrhea | 5 (14.1) | 20 (3.8) |  | 19 (7.0) | 14 (13.5) | 1422 (6.8) |
| **Number of symptoms, median (IQR)** | | 5 (3–7) | 2 (0–4) |  | 2 (0–5) | 4 (2–6) | 1 (0–3) |
|  | Zero symptoms, no. (%) | 4 (9.8) | 137 (30.8) |  | 117 (38.6) | 9 (6.1) | 8777 (46.9) |
| **Outcomes, no. (%)** | |  |  |  |  |  |  |
|  | Sought medical treatment | 28 (77.0) | 267 (52.8) |  | 133 (39.8) | 77 (78.7) | 7306 (36.8) |
|  | Hospitalized | 2 (8.8) | 14 (3.1) |  | 2 (0.6) | 18 (16.2) | 21 (0.1) |
|  | Died | 0 (0.0) | 0 (0.0) |  | 1 (0.3) | 2 (3.0) | 3 (<0.1) |
| *Summary statistics are presented as unweighted counts, weighted percentages, and weighted continuous statistics. | | | | | | | |

| **Table S6. Presenting symptoms and outcomes by SARS-CoV-2 classification group, among adults 20–49 years old.** | | | | | | | |
| --- | --- | --- | --- | --- | --- | --- | --- |
| **Characteristic*** | | **Known SARS-CoV-2 status** | |  | **Unknown SARS-CoV-2 status** | | |
|  |  | **Confirmed or probable infection (N = 147)** | **Negative (N = 1791)** |  | **With close contact (N = 371)** | **No close contact** | |
|  |  |  |  |  |  | **With pneumonia/ lung infection (N = 84)** | **No pneumonia/ lung infection (N = 25180)** |
| **Sex, no. (%)** | |  |  |  |  |  |  |
|  | Female | 65 (43.0) | 706 (39.0) |  | 221 (59.0) | 57 (71.7) | 13279 (52.7) |
|  | Male | 82 (57.0) | 1085 (61.0) |  | 150 (41.0) | 27 (28.3) | 11901 (47.3) |
| **Symptoms, no. (%)** | |  |  |  |  |  |  |
|  | Fever | 137 (92.3) | 1394 (78.6) |  | 212 (54.1) | 71 (83.0) | 12867 (50.7) |
|  | Cough | 107 (77.2) | 792 (40.8) |  | 119 (30.1) | 62 (70.0) | 7235 (27.2) |
|  | Headaches | 103 (71.3) | 1016 (58.1) |  | 131 (34.6) | 54 (64.3) | 8569 (33.5) |
|  | Runny nose/congestion/sneezing | 96 (67.8) | 799 (41.5) |  | 121 (33.4) | 51 (58.3) | 7349 (27.1) |
|  | Chills | 100 (67.6) | 605 (32.8) |  | 96 (27.1) | 41 (48.9) | 5363 (21.4) |
|  | Sore throat | 78 (57.6) | 383 (20.7) |  | 74 (20.4) | 38 (41.8) | 3503 (12.6) |
|  | Muscle pain | 69 (49.3) | 467 (24.4) |  | 55 (15.4) | 41 (47.4) | 3603 (13.6) |
|  | Difficulty breathing | 58 (44.2) | 174 (9.3) |  | 36 (9.9) | 38 (44.8) | 1446 (5.1) |
|  | Loss of taste/smell | 54 (38.5) | 205 (10.2) |  | 30 (8.6) | 19 (19.8) | 1678 (5.5) |
|  | Vomiting | 29 (22.6) | 124 (5.9) |  | 18 (4.9) | 17 (21.3) | 1478 (5.4) |
|  | Diarrhea | 19 (15.8) | 97 (4.9) |  | 14 (3.6) | 10 (14.3) | 1440 (5.3) |
| **Number of symptoms, median (IQR)** | | 6 (4–9) | 3 (2–5) |  | 2 (0–4) | 5 (2–7) | 1 (0–4) |
|  | Zero symptoms, no. (%) | 5 (5.5) | 261 (14.8) |  | 129 (38.5) | 6 (6.9) | 10634 (43.0) |
| **Outcomes, no. (%)** | |  |  |  |  |  |  |
|  | Sought medical treatment | 129 (86.8) | 1276 (68.0) |  | 180 (47.1) | 71 (83.1) | 10463 (39.7) |
|  | Hospitalized | 30 (21.1) | 44 (2.6) |  | 2 (4.7) | 14 (17.2) | 102 (0.3) |
|  | Died | 2 (0.7) | 2 (0.1) |  | 1 (0.4) | 0 (0.0) | 5 (<0.1) |
| *Summary statistics are presented as unweighted counts, weighted percentages, and weighted continuous statistics. | | | | | | | |

| **Table S7. Presenting symptoms and outcomes by SARS-CoV-2 classification group, among adults ≥50 years old.** | | | | | | | |
| --- | --- | --- | --- | --- | --- | --- | --- |
| **Characteristic*** | | **Known SARS-CoV-2 status** | |  | **Unknown SARS-CoV-2 status** | | |
|  |  | **Confirmed or probable infection (N = 97)** | **Negative (N = 822)** |  | **With close contact (N = 103)** | **No close contact** | |
|  |  |  |  |  |  | **With pneumonia/ lung infection (N = 95)** | **No pneumonia/ lung infection (N = 8793)** |
| **Sex, no. (%)** | |  |  |  |  |  |  |
|  | Female | 36 (38.8) | 285 (33.3) |  | 46 (43.1) | 59 (63.1) | 4111 (46.7) |
|  | Male | 61 (61.2) | 537 (66.7) |  | 57 (56.9) | 36 (36.9) | 4682 (53.3) |
| **Symptoms, no. (%)** | |  |  |  |  |  |  |
|  | Fever | 93 (96.9) | 688 (84.6) |  | 76 (72.1) | 84 (93.1) | 5274 (59.9) |
|  | Cough | 83 (88.5) | 451 (51.2) |  | 52 (52.8) | 67 (72.8) | 3416 (37.8) |
|  | Headaches | 72 (77.8) | 496 (58.4) |  | 51 (51.7) | 71 (76.5) | 3692 (42.9) |
|  | Difficulty breathing | 63 (74.8) | 162 (19.0) |  | 20 (21.6) | 55 (60.5) | 1077 (10.8) |
|  | Runny nose/congestion/sneezing | 67 (71.3) | 387 (45.7) |  | 43 (38.5) | 51 (52.7) | 2967 (32.6) |
|  | Chills | 64 (70.4) | 338 (39.4) |  | 45 (43.3) | 57 (63.4) | 2506 (28.2) |
|  | Muscle pain | 60 (66.1) | 396 (44.4) |  | 37 (42.8) | 64 (69.5) | 2693 (29.9) |
|  | Sore throat | 58 (61.6) | 233 (27.4) |  | 27 (24.3) | 43 (47.0) | 1495 (15.6) |
|  | Loss of taste/smell | 42 (50.4) | 119 (13.3) |  | 14 (7.4) | 31 (31.7) | 872 (8.7) |
|  | Vomiting | 22 (23.7) | 73 (8.5) |  | 10 (5.8) | 18 (18.4) | 646 (6.9) |
|  | Diarrhea | 16 (14.1) | 73 (8.3) |  | 7 (3.7) | 21 (22.0) | 613 (6.3) |
| **Number of symptoms, median (IQR)** | | 8 (4–9) | 4 (2–6) |  | 4 (2–5) | 6 (4–8) | 2 (0–5) |
|  | Zero symptoms, no. (%) | 1 (0.7) | 60 (7.7) |  | 19 (19.5) | 1 (0.4) | 2843 (32.2) |
| **Outcomes, no. (%)** | |  |  |  |  |  |  |
|  | Sought medical treatment | 91 (95.7) | 699 (84.3) |  | 69 (64.2) | 88 (96.0) | 4520 (50.4) |
|  | Hospitalized | 40 (42.9) | 72 (8.0) |  | 9 (9.8) | 25 (25.9) | 116 (1.3) |
|  | Died | 13 (14.7) | 23 (2.8) |  | 4 (3.3) | 10 (10.3) | 29 (0.4) |
| *Summary statistics are presented as unweighted counts, weighted percentages, and weighted continuous statistics. | | | | | | | |

| **Table S8. Characteristics of participants with confirmed or probable COVID-19, by disease severity.** | | | | |
| --- | --- | --- | --- | --- |
| **Characteristic*** | | **Outcome** | | **PR for hospitalization or death (95% CI)†** |
|  |  | **Non-severe (N = 233)** | **Hospitalized or died (N = 81)** |  |
| **Age, median (IQR)** | | 30 (20–47) | 50 (28–65) |  |
|  | <5 | 11 (4.6) | 5 (5.1) | 1.02 (0.23–4.65) |
|  | 5 to 9 | 14 (6.7) | 3 (3.6) | 0.58 (0.19–1.77) |
|  | 10 to 14 | 13 (4.8) | 1 (0.8) | 0.21 (0.01–4.13) |
|  | 15 to 19 | 22 (8.3) | 1 (2.5) | 0.36 (0.03–4.35) |
|  | 20 to 29 | 49 (23.5) | 13 (15.0) | 0.67 (0.28–1.63) |
|  | 30 to 39 | 35 (14.3) | 10 (15.4) | Reference |
|  | 40 to 49 | 33 (14.8) | 7 (7.3) | 0.54 (0.25–1.16) |
|  | 50 to 59 | 26 (11.4) | 13 (16.6) | 1.23 (0.69–2.18) |
|  | 60 to 69 | 20 (9.0) | 13 (18.7) | 1.53 (0.79–2.99) |
|  | ≥70 | 10 (2.5) | 15 (14.9) | **2.40 (1.16–4.98)** |
| **Sex, no. (%)** | |  |  |  |
|  | Female | 101 (42.5) | 35 (43.8) | Reference |
|  | Male | 132 (57.5) | 46 (56.2) | 0.96 (0.59–1.56) |
| **Symptoms, no. (%)** | |  |  |  |
|  | Fever | 213 (91.5) | 80 (98.9) | --- |
|  | Chills | 163 (74.1) | 50 (57.9) | 0.60 (0.34–1.05) |
|  | Cough | 168 (75.7) | 69 (89.3) | **2.66 (1.15–6.16)** |
|  | Runny nose/congestion/sneezing | 160 (72.4) | 44 (53.0) | 0.55 (0.29–1.05) |
|  | Headaches | 153 (68.9) | 60 (72.9) | 1.15 (0.66–2.03) |
|  | Sore throat | 120 (56.6) | 44 (55.1) | 0.96 (0.36–2.56) |
|  | Muscle pain | 104 (47.8) | 37 (48.7) | 1.03 (0.49–2.17) |
|  | Difficulty breathing | 94 (46.9) | 49 (60.9) | 1.51 (0.73–3.12) |
|  | Loss of taste/smell | 78 (38.5) | 28 (36.6) | 0.94 (0.44–2.01) |
|  | Vomiting | 50 (24.8) | 28 (34.2) | 1.38 (0.70–2.75) |
|  | Diarrhea | 43 (19.5) | 13 (14.0) | 0.74 (0.40–1.38) |
| **Number of symptoms, median (IQR)** | | 6 (4–9) | 6 (4–9) | 1.00 (0.90–1.12) |
|  | Zero symptoms, no. (%) | 11 (6.0) | 0 (0.0) | --- |
| **Any concurrent health conditions, no. (%)** | | --- | 11 (16.3) | --- |
| *Summary statistics are presented as unweighted counts, and weighted percentages. †Prevalence ratios (PR) did not adjust for other variables given a low event-per-variable ratio. Confidence intervals not crossing the null value of 1 are bolded. | | | | |

| **Table S9. Chronic conditions amongst hospitalized or deceased individuals, by SARS-CoV-2 classification group.** | | | | | | | |
| --- | --- | --- | --- | --- | --- | --- | --- |
| **Characteristic*** | | **Known SARS-CoV-2 history** | |  | **Unknown SARS-CoV-2 history** | | |
|  |  | **Confirmed or probable infection** | **Negative** |  | **With close contact** | **No close contact** | |
|  |  |  |  |  |  | **With pneumonia/ lung infection** | **No pneumonia/ lung infection** |
| **Total hospitalizations and deaths, no.** | | 81 | 140 |  | 15 | 171 | 370 |
| **Any chronic health conditions, no. (%)** | | 16 (18.2) | 42 (27.8) |  | 7 (35.2) | 23 (12.9) | 79 (20.1) |
|  | Cancer | 0 (0.0) | 2 (2.0) |  | 1 (5.0) | 1 (0.7) | 2 (1.1) |
|  | Diabetes | 3 (3.5) | 4 (2.8) |  | 1 (0.9) | 0 (0.0) | 7 (2.0) |
|  | Heart disease or high blood pressure | 7 (9.2) | 23 (13.3) |  | 5 (29.3) | 8 (4.8) | 42 (9.3) |
|  | Immune compromised | 1 (0.9) | 2 (2.5) |  | 0 (0.0) | 1 (0.9) | 2 (0.4) |
|  | Kidney disease | 1 (0.9) | 7 (4.9) |  | 0 (0.0) | 3 (1.5) | 20 (4.9) |
|  | Liver disease | 0 (0.0) | 6 (5.1) |  | 0 (0.0) | 2 (1.1) | 7 (1.6) |
|  | Lung disease | 13 (13.0) | 6 (4.3) |  | 2 (11.1) | 17 (9.5) | 8 (2.8) |
|  | Neurological/brain disease | 1 (0.9) | 2 (0.8) |  | 0 (0.0) | 3 (0.6) | 7 (1.8) |
| **Pregnant, no. / total No. (%)†** | | 0 / 14 (0.0) | 2 / 27 (6.3) |  | 0 / 4 (0.0) | 0 / 15 (0.0) | 7 / 77 (7.6) |
| **Recently gave birth (last 6 weeks), no. / total No. (%)†** | | 1 / 14 (5.0) | 2 / 27 (6.4) |  | 0 / 4 (0.0) | 0 / 15 (0.0) | 1 / 77 (1.4) |
| *Summary statistics are presented as unweighted counts, weighted percentages, and weighted continuous statistics. †Statistics reported from a denominator of females aged 15–49. | | | | | | | |

| **Table S10. Comparison of study indicators versus GB Department of Health statistics.** | | | | | | | |
| --- | --- | --- | --- | --- | --- | --- | --- |
| **District** | | **SARS-CoV-2 tests administered** | |  | **Positive SARS-CoV-2 tests** | | **Test Positivity, % (95% CI)** |
|  |  | **No.** | **per 1,000 people (95% CI)** |  | **No.** | **per 1,000 people (95% CI)** |  |
| **Astore** | |  |  |  |  |  |  |
|  | Study catchment area | 462 | 61.0 (39.1–82.9) |  | 6 | 0.3 (0.0–0.7) | 0.6 (0.2–1.8) |
|  | Reported by GB DoH | 13722 | 129.8 |  | 443 | 4.2 | 3.2 |
| **Diamer** | |  |  |  |  |  |  |
|  | Study catchment area | 222 | 57.1 (25.1–89.0) |  | 20 | 5.9 (0.0–12.1) | 10.6 (2.7–33.9) |
|  | Reported by GB DoH | 15551 | 49.7 |  | 380 | 1.2 | 2.4 |
| **Ghanche** | |  |  |  |  |  |  |
|  | Study catchment area | 359 | 18.6 (11.6–25.6) |  | 24 | 1.8 (0.0–4.2) | 10.2 (2.9–30.1) |
|  | Reported by GB DoH | 7707 | 52.1 |  | 160 | 1.1 | 2.1 |
| **Kharmang** | |  |  |  |  |  |  |
|  | Study catchment area | 274 | 47.8 (24.6–71.1) |  | 3 | 0.4 (0.0–1.0) | 0.8 (0.1–4.7) |
|  | Reported by GB DoH | 5457 | 92.0 |  | 132 | 2.2 | 2.4 |
| **Nagar** | |  |  |  |  |  |  |
|  | Study catchment area | 587 | 71.2 (58.6–83.8) |  | 27 | 4.1 (1.2–6.9) | 5.9 (3.1–10.9) |
|  | Reported by GB DoH | 6067 | 97.6 |  | 239 | 3.8 | 3.9 |
| **Shigar** | |  |  |  |  |  |  |
|  | Study catchment area | 721 | 70.3 (47.5–93.1) |  | 14 | 1.3 (0.4–2.2) | 1.9 (0.9–3.7) |
|  | Reported by GB DoH | 5128 | 61.4 |  | 177 | 2.1 | 3.5 |
| **Skardu** | |  |  |  |  |  |  |
|  | Study catchment area | 921 | 42.4 (30.8–54.1) |  | 73 | 3.7 (1.4–6.1) | 9.0 (5.8–13.7) |
|  | Reported by GB DoH | 15470 | 53.1 |  | 821 | 2.8 | 5.3 |
| **TOTAL** | |  |  |  |  |  |  |
|  | Study catchment area | 3546 | 47.9 (42.0–53.9) |  | 167 | 2.6 (1.6–3.6) | 5.5 (3.8–8.0) |
|  | Reported by GB DoH | 69102 | 42.3 |  | 2352 | 1.4 | 3.4 |
| CI = Confidence internval; GB DoH = Gilgit-Baltistan Department of Health. | | | | | | | |

# **Appendix 1: Calculation of SARS-CoV-2 testing rates**

To contextualize the low rate of SARS-CoV-2 testing in Gilgit-Baltistan (GB), we compared daily testing rates in GB to other global jurisdictions. Specifically, we compared daily testing rates to those from all of Pakistan, India and Iran (two nations bordering Pakistan), and the United States (for a high-income country comparison).

Daily testing rates in GB were calculated as follows. The cumulative number of SARS-CoV-2 tests conducted in our seven study districts (i.e. Astore, Diamer, Ghanche, Kharmang, Nagar, Shigar, and Skardu) between March 1, 2020–June 13, 2021 was provided by the GB Department of Health. This date range corresponds to the beginning of the recall period specified in our questionnaire, up until the day prior to the first data collection visits. Population denominators for the seven study districts in 2021 were also provided by the GB Department of Health, determined through their Expanded Program on Immunization. To calculate daily testing rates, the cumulative number of SARS-CoV-2 tests was divided by the number of days in the recall period (i.e. 470 days), and then further divided by the corresponding population denominator.

Daily testing rates in Pakistan, India, Iran, and the United States were calculated as follows. Daily testing rates were compiled from OurWorldInData.org, which in turn compiled these data from national public health bodies worldwide. Data sources include the Government of Pakistan (March 18, 2020–June 13, 2021), Government of Iran (April 12, 2020–June 13, 2021), Indian Council of Medical Research (March 20, 2020–June 13, 2021), and the United States Department of Health & Human Services (March 8, 2020–June 13, 2021). For each country, the 7-day rolling average of daily testing rates per 1,000 people was reported. For each country, the mean daily testing rate within the respective periods was calculated and compared to the daily testing rate in GB as calculated above.

#
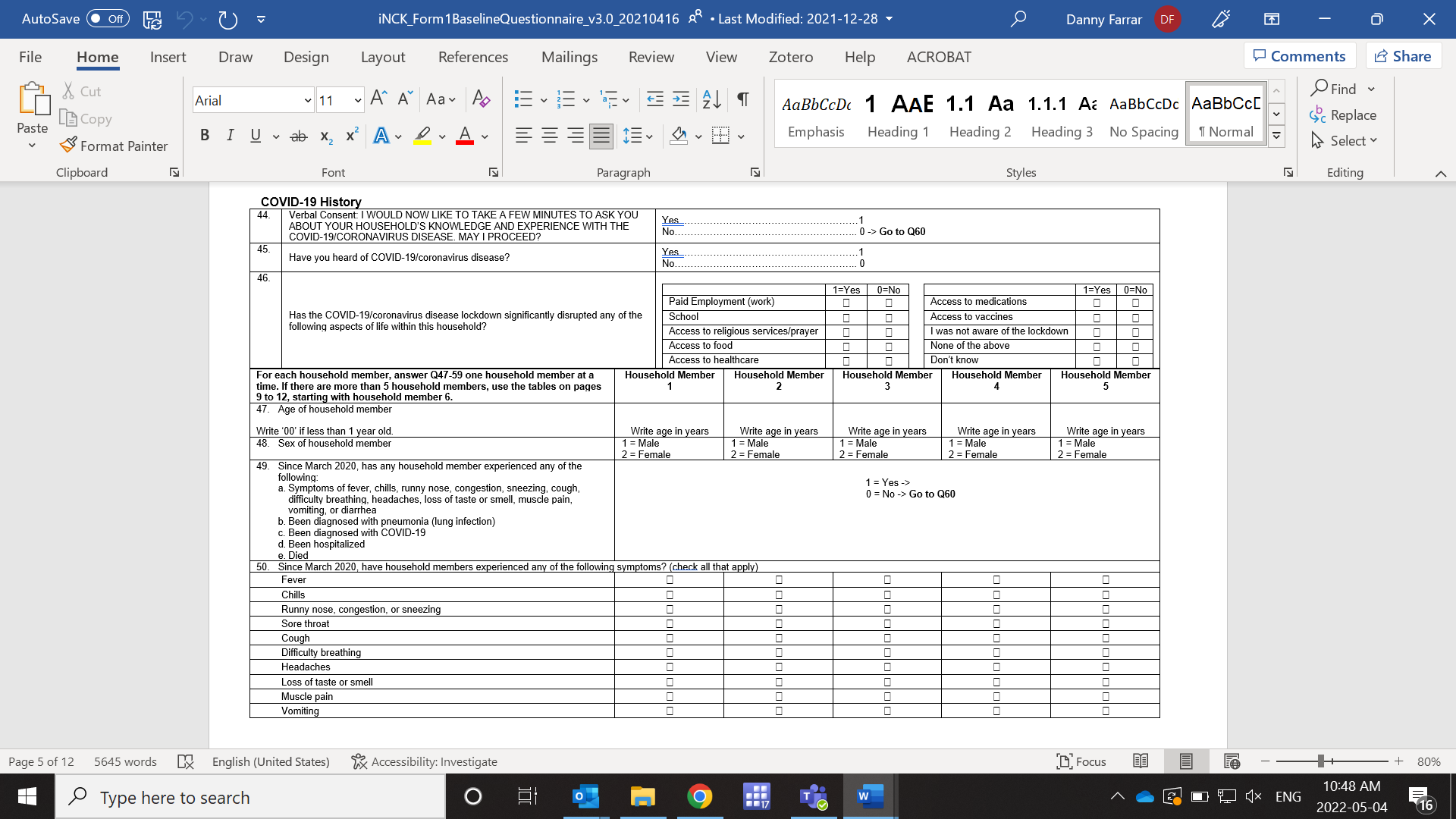
**Appendix 2: COVID-19 study data collection form**


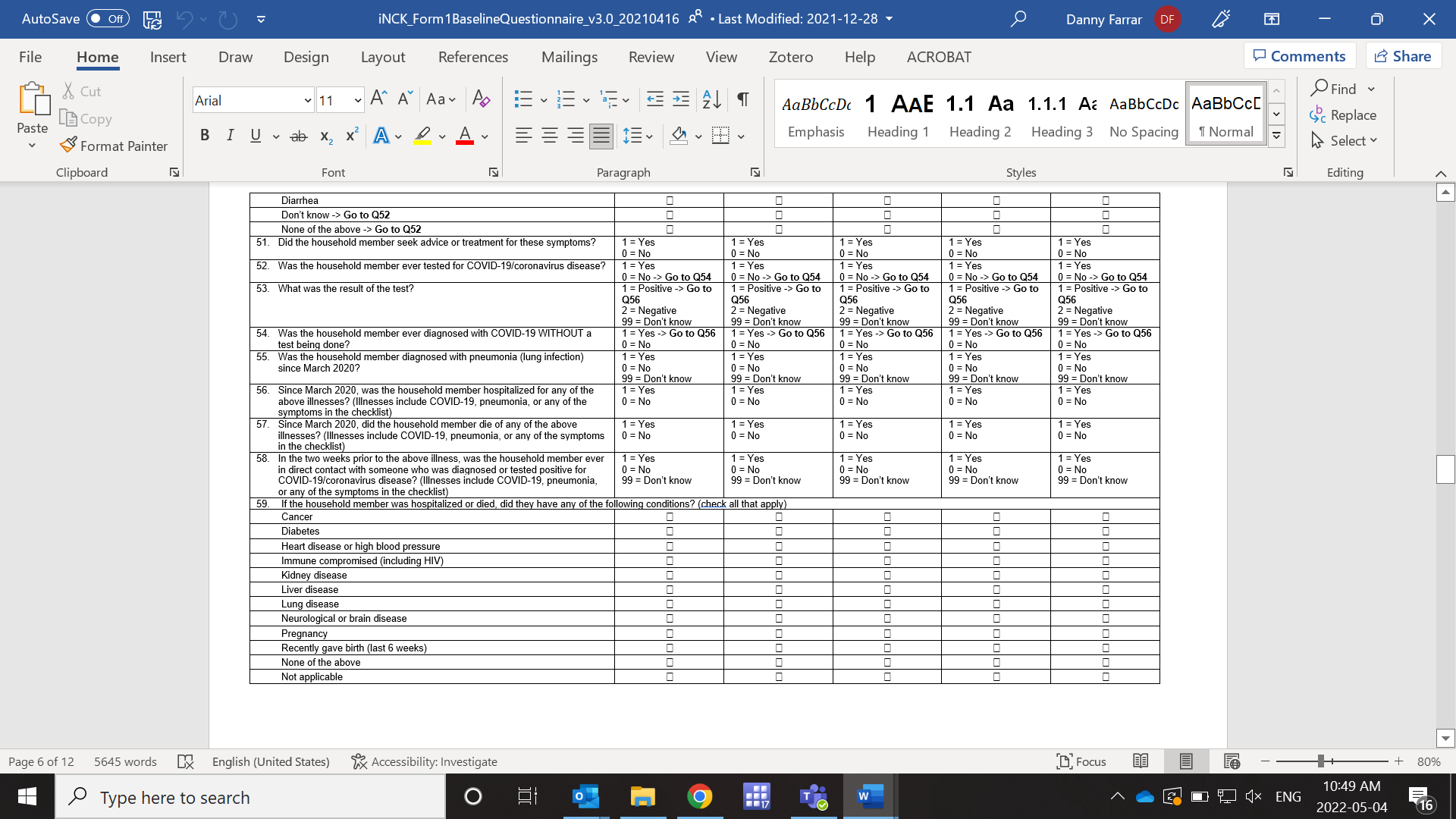


# **Appendix 3: Machine learning model selection and validation**

*Overview*

To identify infected but untested individuals with SARS-CoV-2 infection, we aimed to: a) develop a predictive model of prior SARS-CoV-2 infection among individuals with known SARS-CoV-2 history (i.e. those with confirmed/probable infections vs. negative SARS-CoV-2 tests); and b) apply this model to untested individuals to predict prior SARS-CoV-2 infection.

To develop a predictive model, we conducted a machine learning (ML) analysis among 3,577 individuals with known SARS-CoV-2 history, including 314 with confirmed/probable infection and 3,263 with negative SARS-CoV-2 tests. A summary of the complete ML workflow is described in Figure S2.

*Variable selection*

Covariates of interest were defined at the time of questionnaire development, and were based on literature review of COVID-19 related symptoms and availability of demographic and close contact information. Covariates included age (collected as a discrete variable), sex, a series of 11 binary presenting symptoms, any healthcare seeking (vs. none), a composite variable denoting either hospitalization or death (vs. neither), and a variable denoting either a household or external close contact with positive COVID-19 diagnosis in the two weeks prior to any symptoms (vs. no contact). To account for potential nonlinear associations with age (measured in years), the association with age was modelled using a restricted cubic spline with four knots at evenly spaced percentiles^[[1]](#footnote-1)^ (i.e. 5^th^, 35^th^, 65^th^, and 95^th^ percentiles). The eleven symptoms collected included chills, cough, diarrhea, difficulty breathing, fever, headaches, loss of taste or smell, muscle pain, runny nose/congestion/sneezing, sore throat, and vomiting. Early in the analysis, the ‘close contact’ variable was removed as its feature importance was orders of magnitude greater than any other covariate of interest. We interpreted this association as indicating collider bias, given multiple positive SARS-CoV-2 tests or healthcare worker diagnoses sometimes occurred within the same household (i.e. and were close contacts of one another). In lieu of this variable, we included a covariate denoting the Z-score normalized rate of village-level confirmed/probable infections. This rate was calculated as the number of confirmed/probable infections within the village of residence, divided by the number of participants with complete data collection in the village, then standardized to a mean of zero and standard deviation of one.

Data regarding age, sex, and outcomes (i.e., healthcare seeking, hospitalization, and death) were complete for all participants. Data collection for the 11 presenting symptoms was conducted using a checklist, whereby absence of an affirmative response was assumed to indicate a null response (i.e., absence of the specific symptom).

*Model selection and validation*

ML analyses were conducted using Python (version 3.11.0) and the scikit-learn library (version 1.3.0). The data were first split into training and testing sets using a random 80/20 split. The test set, comprising 20% of observations, was reserved from the outset of the ML analysis and later used to calculate validation statistics for all trained models.

We trained models using four common ML algorithms, including logistic regression (LR), support vector machines (SVM), random forest (RF), and extreme gradient boosting (XGB). We also trained a fifth model using a voting classifier, which trained on an ensemble of the LR, SVM, RF, and XGB methods. The voting classifier used soft voting (i.e. based on the sum of predicted probabilities across all four ensemble methods) and equal importance weights. These algorithms were selected because they accommodated: a) the binary outcome of interest; and b) the inclusion of survey weights in their native coding, in order to account for the selection of villages and households in the sampling design. To optimize predictive performance of the models and minimize the loss functions, we conducted hyperparameter tuning for the LR, SVM, RF, and XGB algorithms using 10-fold grid search cross validation. Specific hyperparameter values were defined *a priori* and were based on literature review, and are listed in Appendix Table 1.

To assess model performance and calculate validation statistics, we then conducted bootstrap validation for each of the five methods using the tuned hyperparameters. The training set was not partitioned into development and validation sets, and validation statistics were instead calculated using predicted classes and probabilities from the reserved testing set. One thousand bootstrap replicates were analyzed for each algorithm, each containing a random 70% of the training data. We further observed class imbalance (i.e. 8.8% confirmed/probable infections and 91.2% negative SARS-CoV-2 tests in the full dataset), and therefore randomly undersampled the majority class to achieve a 4:1 majority:minority ratio. Bootstrapped models were therefore fit to sets including roughly 160–200 confirmed/probable infections and 640–800 negative SARS-CoV-2 tests.

Validation statistics with 95% confidence intervals were calculated by applying bootstrapped predicted classes and probabilities to the reserved test set. We calculated area under the receiver operating characteristic curve (auROC), area under the precision-recall curve (auPRC), sensitivity (recall), specificity, F1 score, and accuracy. The best algorithm was selected on the basis of the highest auROC score, while sensitivity and specificity values were selected as those which maximized Youden’s J statistic. Validation statistics at the optimal sensitivity and specificity values for all five algorithms are presented in Appendix Table 2. We visualized further validation statistics using ROC curves, PRC curves, calibration curves, and confusion matrices (Appendix Figures 2–6). Calibration curves were presented with untransformed values, as well as transformed values using Platt scaling of the predicted probabilities.

Finally, additional validation statistics for the selected XGBoost model are presented in Appendix Table 3, at pre-specified levels of sensitivity and specificity. We also assessed feature importance of the selected XGBoost algorithm (Appendix Figure 7).


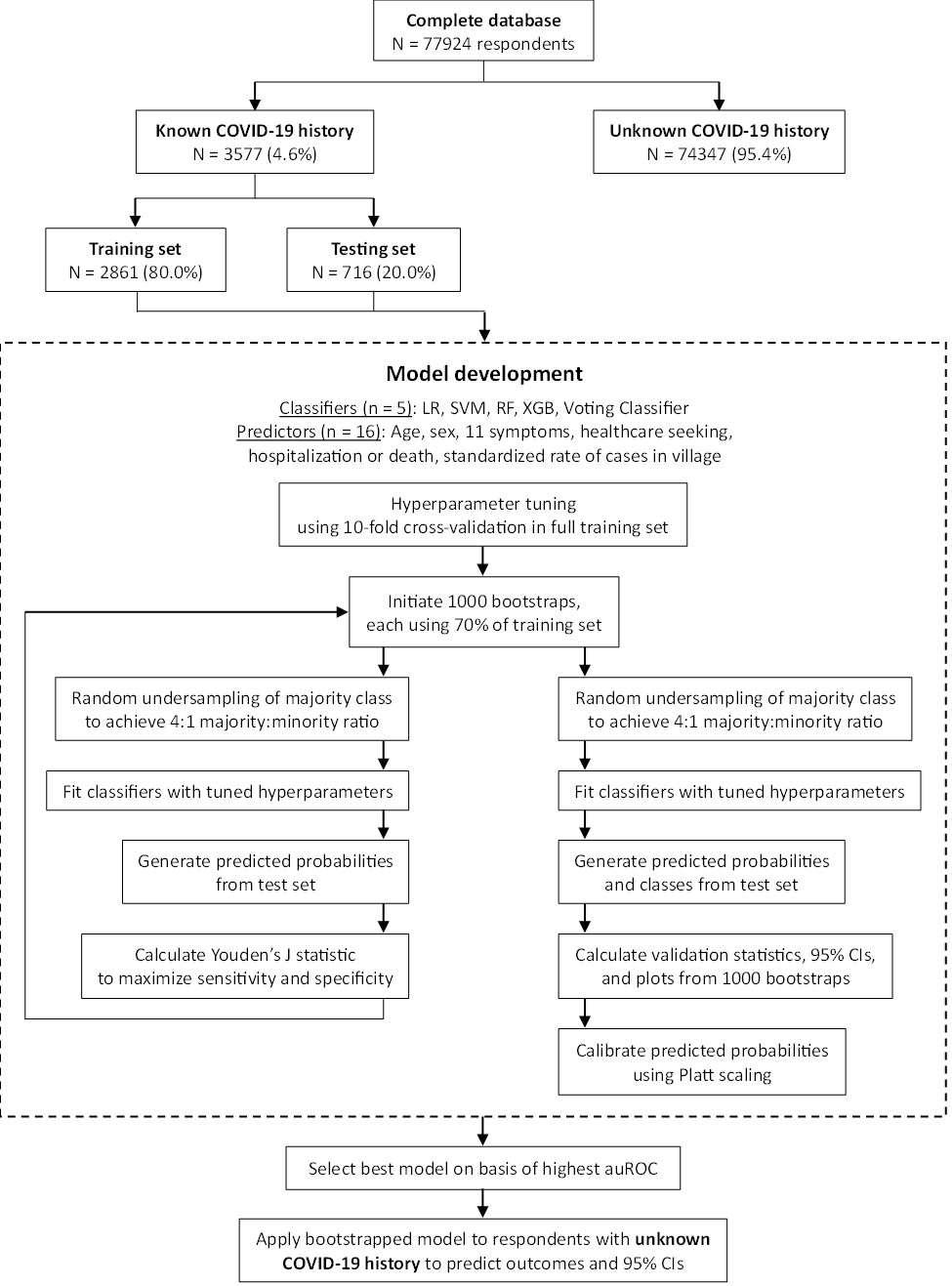


## **Figure S2.** Workflow diagram for machine learning analysis.

auROC=Area under the receiver operating characteristic curve; CI=Confidence interval; LR=Logistic regression; RF=Random forest; SVM=Support vector machines; XGB=eXtreme Gradient Boosting

## **Table S11.** Hyperparameter values included in the 10-fold grid search cross validation.

| **Model** | **Hyperparameter** | **Possible values** | **Selected value** |
| --- | --- | --- | --- |
| Logistic regression  (LR) | C | [0.0001, 0.0005, 0.001, 0.005, 0.01, 0.05, 0.1, 0.5, 1, 5, 10] | 5 |
|  | penalty | [‘l1’, ‘l2’, ‘elasticnet’] | ‘l1’ |
|  | solver | [‘lbfgs’, ‘liblinear’, ‘saga’] | ‘liblinear’ |
| Support vector machines  (SVM) | C | [0.1, 1, 10, 100, 1000] | 100 |
|  | gamma | [0.0001, 0.001, 0.01, 0.1, 1] | 0.01 |
|  | kernel | [‘linear’, ‘rbf’] | ‘rbf’ |
| Random forest  (RF) | max_depth | [4, 5, 6, 7, 8, None] | 8 |
|  | max_features | [‘auto’, ‘sqrt’, ‘log2’] | ‘auto’ |
|  | min_samples_split | [2, 4, 8] | 8 |
|  | n_estimators | [50, 100, 250, 500, 750, 1000] | 1000 |
| Extreme gradient boosting  (XGB) | gamma | [0, 0.1, 1] | 1 |
|  | learning_rate | [0.01, 0.05, 0.1, 0.3] | 0.1 |
|  | max_depth | [2, 4, 6, 8] | 2 |
|  | n_estimators | [50, 100, 300, 500] | 300 |
|  | reg_alpha | [0, 0.1, 1] | 1 |
|  | reg_lambda | [0, 0.1, 1] | 0.1 |

| **Table S12. Validation statistics and bootstrapped 95% confidence intervals for each trained ML algorithm.** | | | | | | |
| --- | --- | --- | --- | --- | --- | --- |
| **Algorithm** | **auROC** | **auPRC** | **Sensitivity**  **(Recall)** | **Specificity** | **F1-score** | **Accuracy** |
| XGBoost | 0.919  (0.904–0.930) | 0.664  (0.578–0.729) | 0.813  (0.747–0.853) | 0.877  (0.849–0.903) | 0.563  (0.511–0.610) | 0.871  (0.844–0.893) |
| Random forest | 0.899  (0.885–0.913) | 0.598  (0.511–0.690) | 0.833  (0.780–0.881) | 0.848  (0.815–0.875) | 0.526  (0.480–0.568) | 0.846  (0.817–0.870) |
| Logistic regression | 0.886  (0.870–0.899) | 0.633  (0.573–0.684) | 0.795  (0.724–0.851) | 0.875  (0.841–0.902) | 0.551  (0.492–0.605) | 0.867  (0.835–0.892) |
| Support vector machines | 0.884  (0.851–0.908) | 0.617  (0.521–0.694) | 0.778  (0.675–0.847) | 0.860  (0.817–0.901) | 0.518  (0.456–0.581) | 0.851  (0.814–0.885) |
| Voting classifier | 0.908  (0.894–0.920) | 0.664  (0.579–0.729) | 0.816  (0.757–0.855) | 0.875  (0.848–0.900) | 0.561  (0.516–0.603) | 0.869  (0.844–0.889) |
| auROC=Area under the receiver operating curve; auPRC=Area under the precision-recall curve. Values represent bootstrapped means and 95% confidence intervals. | | | | | | |


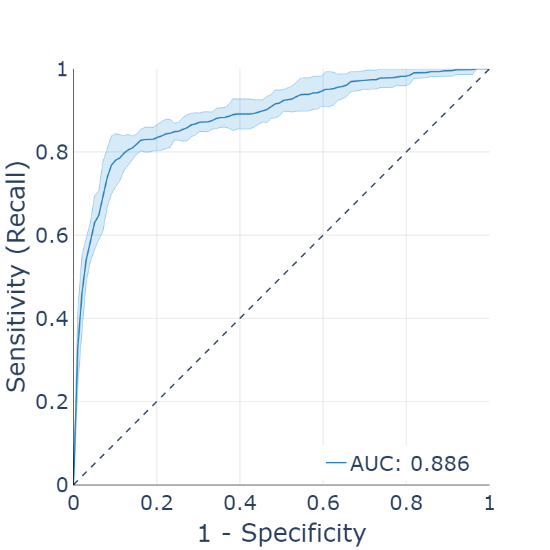

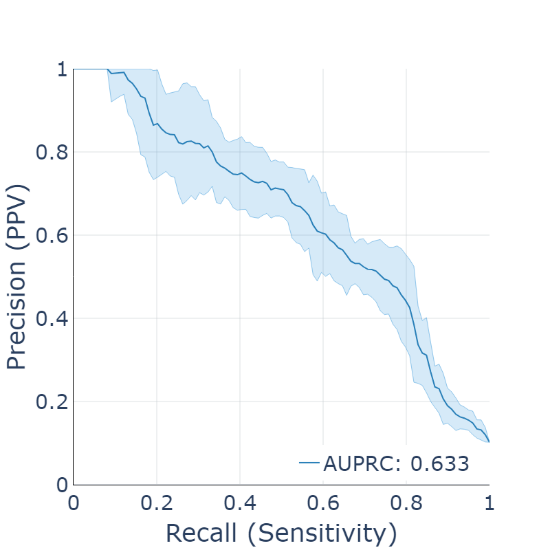

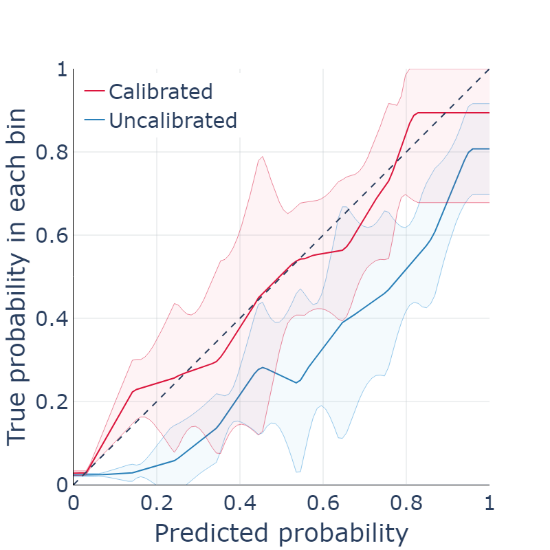


## **Figure S3.** Receiver operating characteristic curve (ROC; left), precision-recall curve (PRC; centre), and calibration curve for logistic regression model.


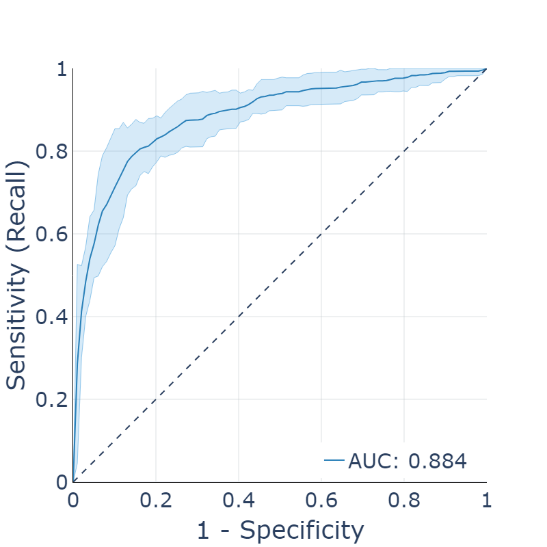

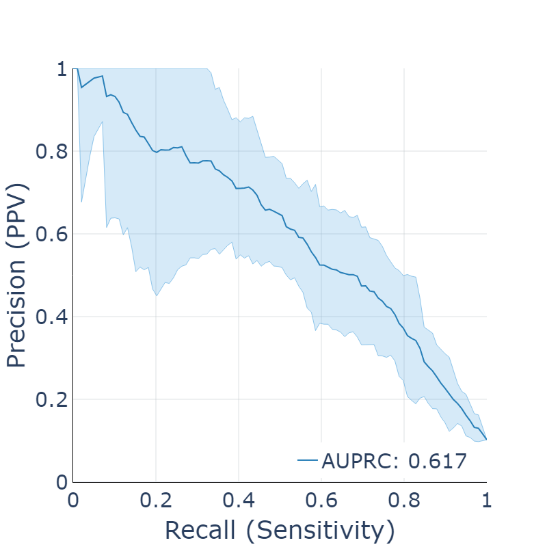

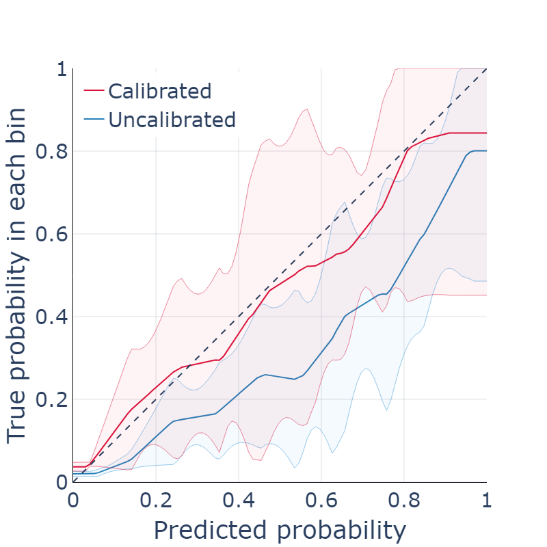


## **Figure S4.** Receiver operating characteristic curve (ROC; left), precision-recall curve (PRC; centre), and calibration curve for support vector machine model.


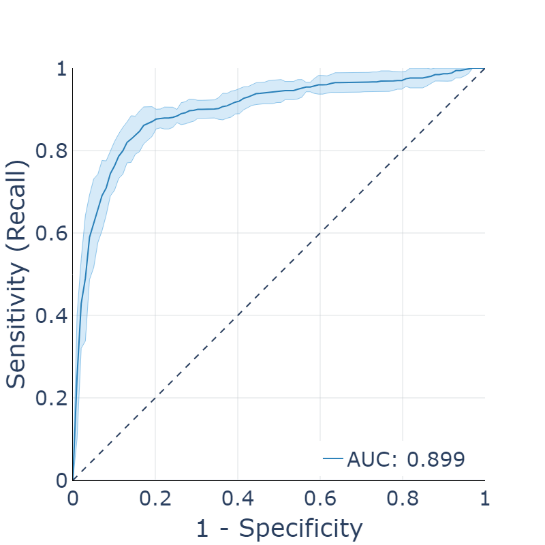

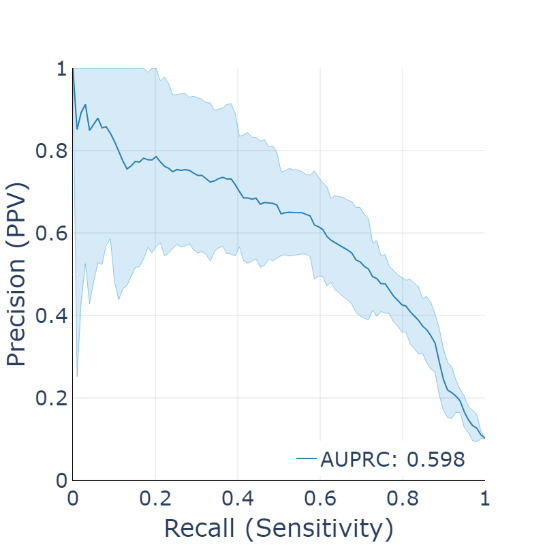

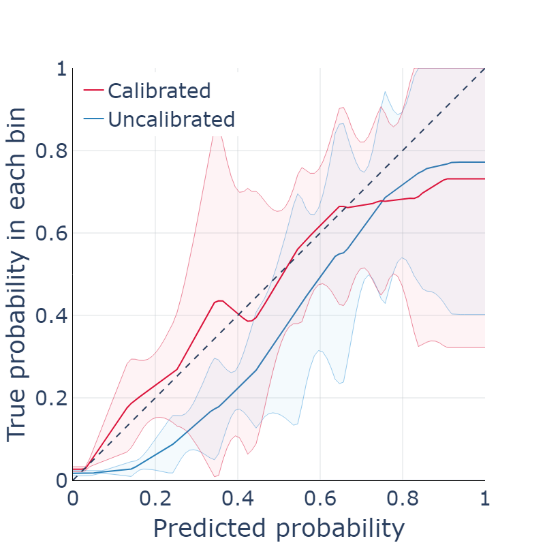


## **Figure S5.** Receiver operating characteristic curve (ROC; left), precision-recall curve (PRC; centre), and calibration curve for random forest model.


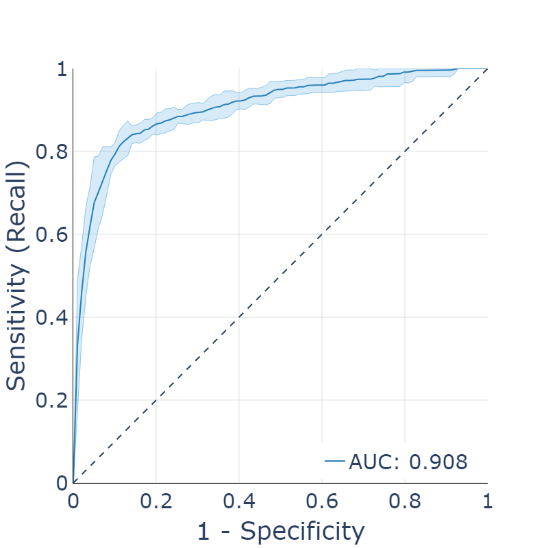

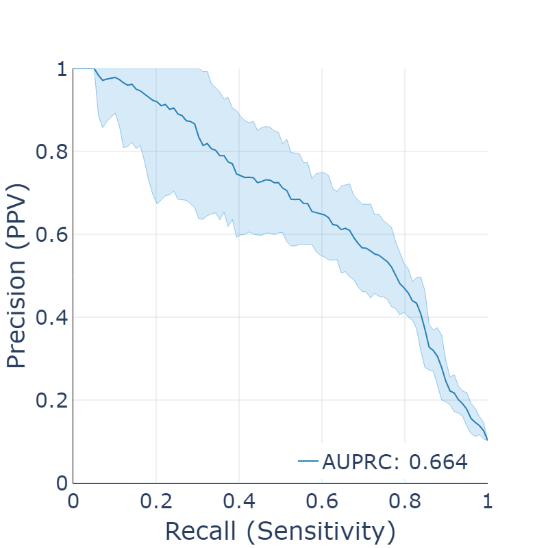

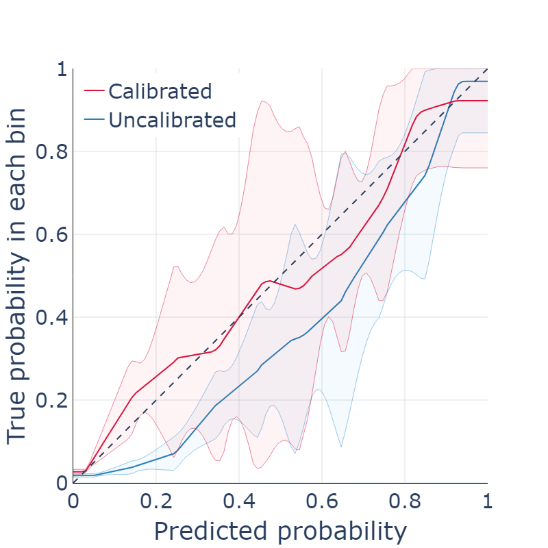


## **Figure S6.** Receiver operating characteristic curve (ROC; left), precision-recall curve (PRC; centre), and calibration curve for voting classifier ensemble model.

| Logistic regression | | Actual Class | |
| --- | --- | --- | --- |
|  |  | Positive (1) | Negative (0) |
| Predicted Class | Positive (1) | **TP** 62.0 (56.4–66.3) | **FP** 85.4 (67.0–108.7) |
|  | Negative (0) | **FN** 15.9 (11.6–21.5) | **TN** 599.1 (575.8–617.5) |
|  |  |  |  |
| Support vector machines | | Actual Class | |
|  |  | Positive (1) | Negative (0) |
| Predicted Class | Positive (1) | **TP** 60.6 (52.6–66.0) | **FP** 96.1 (67.5–125.4) |
|  | Negative (0) | **FN** 17.3 (11.9–25.3) | **TN** 588.4 (559.1–617.0) |
|  |  |  |  |
| Random forest | | Actual Class | |
|  |  | Positive (1) | Negative (0) |
| Predicted Class | Positive (1) | **TP** 64.9 (60.7–68.6) | **FP** 104.3 (85.4–126.9) |
|  | Negative (0) | **FN** 13.0 (9.3–17.2) | **TN** 580.2 (557.6–599.1) |
|  |  |  |  |
| XGBoost | | Actual Class | |
|  |  | Positive (1) | Negative (0) |
| Predicted Class | Positive (1) | **TP** 63.4 (58.2–66.5) | **FP** 84.1 (66.6–103.3) |
|  | Negative (0) | **FN** 14.5 (11.4–19.7) | **TN** 600.4 (581.2–618.0) |
|  |  |  |  |
| Voting classifier | | Actual Class | |
|  |  | Positive (1) | Negative (0) |
| Predicted Class | Positive (1) | **TP** 63.6 (59.0–66.6) | **FP** 85.4 (68.6–104.2) |
|  | Negative (0) | **FN** 14.4 (11.3–18.9) | **TN** 599.1 (580.4–615.9) |

## **Figure S7.** Confusion matrices for the five machine learning algorithms.

FN = False negative; FP = False positive; TN = True negative; TP = True positive.

Values represent bootstrapped means and 95% confidence intervals.

| **Table S13. Validation statistics and bootstrapped 95% confidence intervals for the XGBoost model, at varying thresholds of sensitivity (recall) and specificity.** | | | | |
| --- | --- | --- | --- | --- |
| **Threshold** | **Sensitivity**  **(Recall)** | **Specificity** | **F1-score** | **Accuracy** |
| Sensitivity = 0.95 | --- | 0.584  (0.483–0.675) | 0.341  (0.297–0.389) | 0.622  (0.532–0.700) |
| Sensitivity = 0.90 | --- | 0.729  (0.664–0.781) | 0.422  (0.376–0.468) | 0.746  (0.691–0.792) |
| Sensitivity = 0.85 | --- | 0.826  (0.785–0.863) | 0.504  (0.455–0.552) | 0.828  (0.792–0.861) |
| Optimal threshold | 0.813  (0.747–0.853) | 0.877  (0.849–0.903) | 0.563  (0.511–0.610) | 0.871  (0.844–0.893) |
| Specificity = 0.90 | 0.784  (0.701–0.837) | --- | 0.590  (0.534–0.640) | 0.888  (0.866–0.908) |
| Specificity = 0.95 | 0.655  (0.556–0.756) | --- | 0.626  (0.558–0.687) | 0.920  (0.904–0.934) |
| auROC=Area under the receiver operating curve; auPRC=Area under the precision-recall curve. Values represent bootstrapped means and 95% confidence intervals. | | | | |


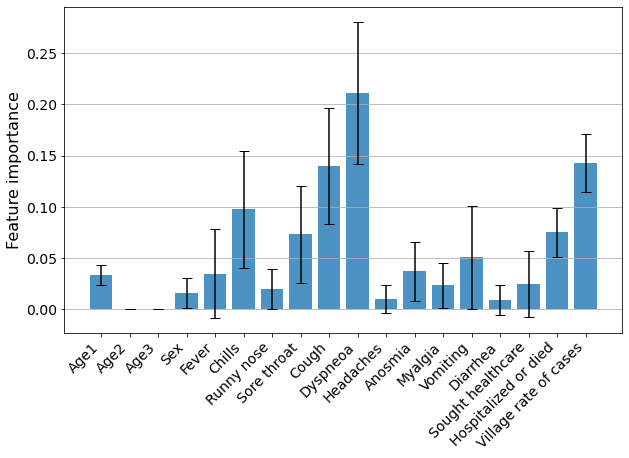


## **Figure S8.** Feature importance coefficients and 95% confidence intervals of the selected XGBoost model.

‘Age1’, ‘Age2’, and ‘Age3’ indicate restricted cubic spline terms for the continuous age variable (measured in years). The spline used four knots at evenly spaced percentiles (i.e. 5th, 35th, 65th, and 95th percentiles). The ‘village rate of cases’ indicates the number of confirmed/probable infections within the participant’s village of residence, divided by the number of participants with complete data collection in the village, then standardized to a mean of zero and standard deviation of one (Z-score normalized rate). All other variables were entered as binary terms.

| **Table S14. Breakdown of confirmed, probable, and possible COVID-19 cases.** | | | | | | | |
| --- | --- | --- | --- | --- | --- | --- | --- |
| **Characteristic** | | **Confirmed SARS-CoV-2, no.** | **Probable COVID** | |  | **Possible COVID-19*** | |
|  |  |  | **HCW diagnosis†, no.** | **ML diagnosis, no. (95% CI)** |  | **Close contact, no. (95% CI)** | **Pneumonia/ lung infection, no. (95% CI)** |
| **Overall** | | 167 | 147 | 1264 (907–1740) |  | 980 (940–1007) | 511 (483–539) |
| **Age group (years)** | |  |  |  |  |  |  |
|  | <1 | 0 | 1 | 49 (19–100) |  | 26 (23–29) | 45 (39–51) |
|  | 1 to 4 | 1 | 14 | 197 (93–405) |  | 112 (93–122) | 157 (141–171) |
|  | 5 to 9 | 2 | 15 | 144 (62–268) |  | 133 (124–139) | 78 (72–84) |
|  | 10 to 14 | 2 | 12 | 115 (51–197) |  | 140 (134–146) | 55 (52–58) |
|  | 15 to 19 | 9 | 14 | 137 (83–223) |  | 142 (135–147) | 35 (33–37) |
|  | 20 to 29 | 33 | 29 | 195 (114–315) |  | 172 (162–179) | 24 (22–26) |
|  | 30 to 39 | 25 | 20 | 112 (64–164) |  | 96 (94–98) | 19 (16–22) |
|  | 40 to 49 | 20 | 20 | 87 (54–128) |  | 72 (68–74) | 23 (21–25) |
|  | 50 to 59 | 30 | 9 | 74 (45–108) |  | 33 (32–35) | 25 (22–27) |
|  | 60 to 69 | 28 | 5 | 87 (56–125) |  | 25 (23–28) | 25 (22–27) |
|  | ≥70 | 17 | 8 | 57 (18–102) |  | 29 (25–32) | 27 (22–31) |
| **Sex** | |  |  |  |  |  |  |
|  | Female | 60 | 76 | 668 (472–933) |  | 502 (482–516) | 265 (253–278) |
|  | Male | 107 | 71 | 596 (404–854) |  | 478 (457–495) | 246 (228–264) |
| **District** | |  |  |  |  |  |  |
|  | Astore | 6 | 8 | 37 (10–77) |  | 83 (78–85) | 43 (38–48) |
|  | Diamer | 20 | 44 | 309 (165–473) |  | 83 (68–95) | 17 (12–22) |
|  | Ghanche | 24 | 12 | 218 (68–432) |  | 123 (112–130) | 86 (83–90) |
|  | Kharmang | 3 | 0 | 3 (0–11) |  | 22 (21–22) | 30 (30–30) |
|  | Nagar | 27 | 46 | 333 (216–446) |  | 208 (196–220) | 72 (61–87) |
|  | Shigar | 14 | 2 | 28 (6–72) |  | 82 (80–82) | 82 (79–85) |
|  | Skardu | 73 | 35 | 336 (206–539) |  | 380 (364–389) | 180 (168–190) |
| *Individuals with close contacts or pneumonia/lung infection who were predicted as positive COVID-19 in the machine learning analysis are not double counted (i.e. only included in the 'machine learning diagnosis' column). †Includes individuals with COVID-19-positive close contacts (and without pneumonia/lung infection) and HCW diagnoses of pneumonia/lung infection (and without positive close contacts). Cases who were predicted as positive COVID-19 in the machine learning analysis are not double counted (i.e. only included in the 'probable' column). | | | | | | | |

1. Harrell J. Regression Modeling Strategies: With Applications to Linear Models, Logistic and Ordinal Regression, and Survival Analysis, 2nd ed. 2015. Cham: Springer International Publishing : Imprint: Springer, 2015 DOI:10.1007/978-3-319-19425-7. [↑](#footnote-ref-1)
